# Supplementary material for: Claulansine F–Donepezil Hybrids as Anti-Alzheimer’s Disease Agents with Cholinergic, Free-Radical Scavenging, and Neuroprotective Activities
Source: Molecules. 2021 Feb 28;26(5):1303. doi: 10.3390/molecules26051303 (PMC7957565; doi:10.3390/molecules26051303)
Supplement: Supplementary file 1 [file molecules-26-01303-s001.pdf]

Claulansine F-Donepezil hybrids as anti-Alzheimer's disease agents with cholinergic, free radical scavenging, and neuroprotective activities

Yingda Zang†, Ke Liu†, Weiping Wang, Chuangjun Li, Jie Ma, Jingzhi Yang, Xinyi Chen, Xiaoliang Wang, Dongming Zhang*

Supplementary Materials

State Key Laboratory of Bioactive Substance and Function of Natural Medicines, Institute of Materia Medica, Chinese Academy of Medical Sciences and Peking Union Medical College, Beijing, 100050, China;

* Correspondence: zhangdm@imm.ac.cn; Tel./Fax: +86-10-63165227

† These authors contributed equally to this work.

Table of Contents

1. ^{1}H NMR and ^{13}C NMR spectra of all final compounds 2-27

2. HPLC analysis of all final compounds 28

1. ^{1}H NMR and ^{13}C NMR spectra of all final compounds

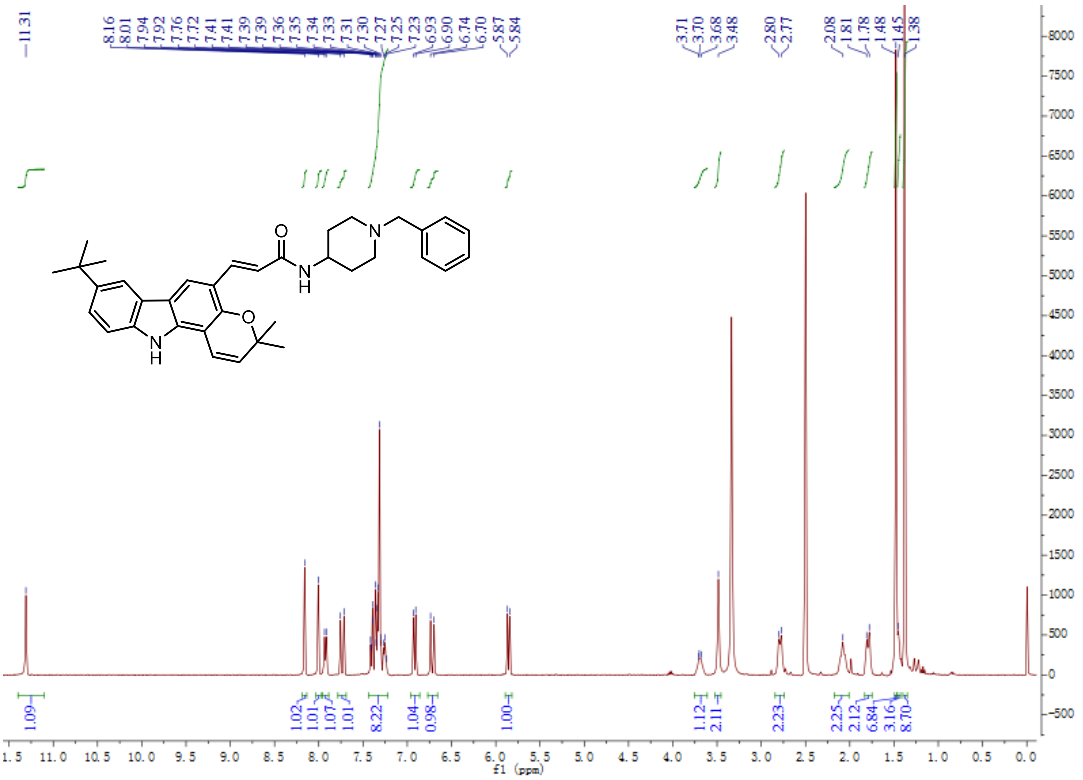

^{1}H NMR (DMSO, 400 MHz) Spectrum of Compound **6aa**

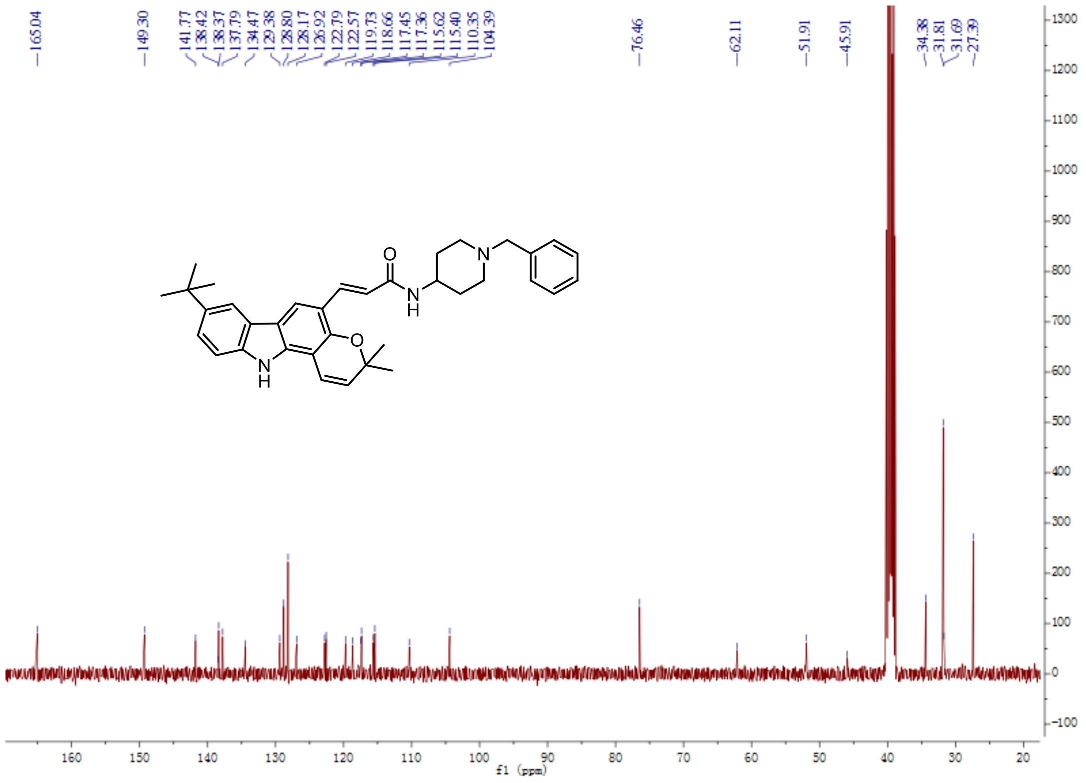

^{13} C NMR (DMSO, 400 MHz) Spectrum of Compound **6aa**

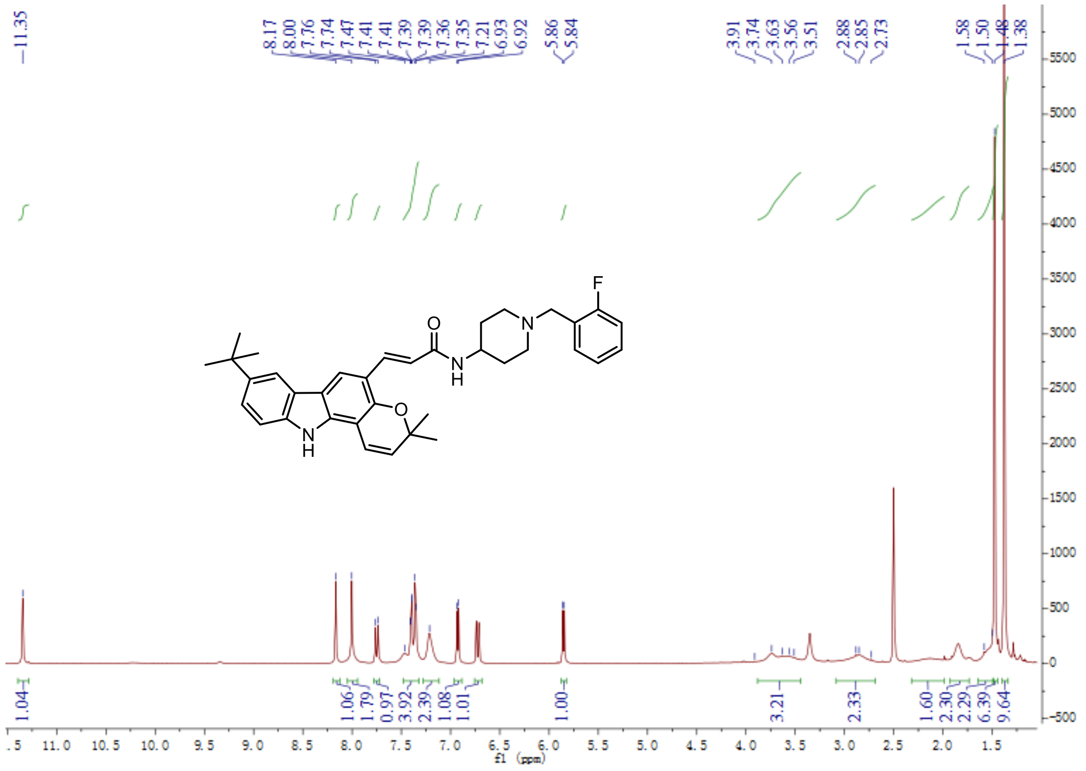

^{1} H NMR (DMSO, 400 MHz) Spectrum of Compound **6ab**

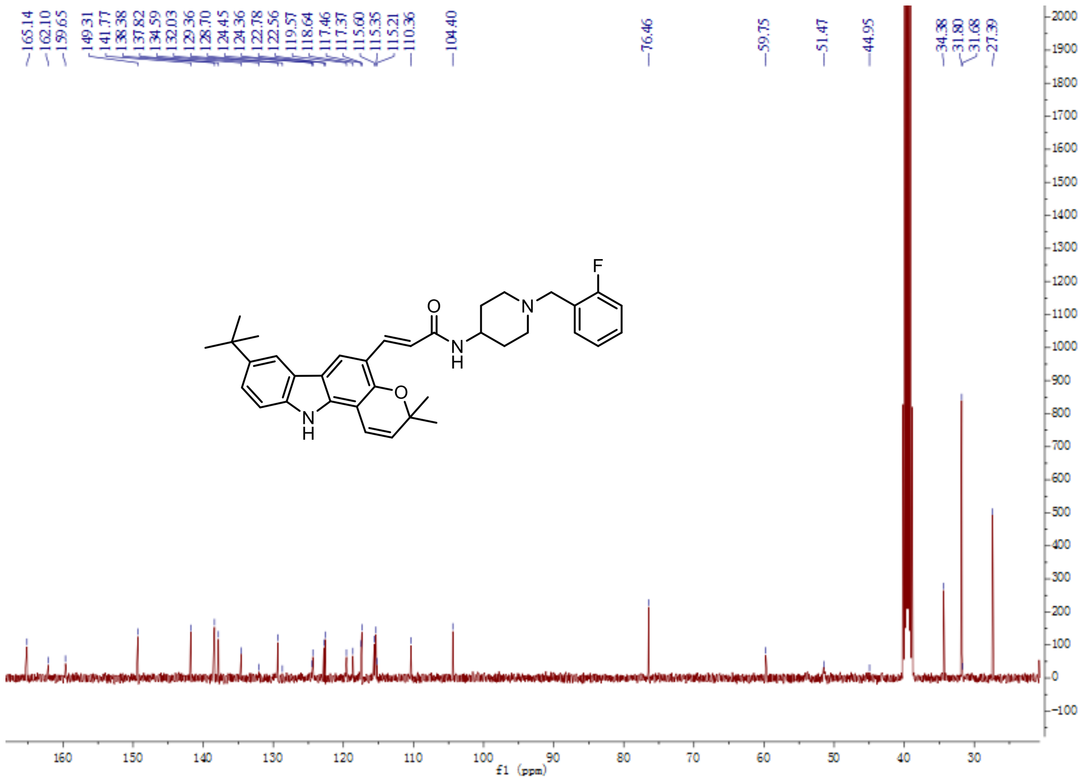

^{13}C NMR (DMSO, 400 MHz) Spectrum of Compound **6ab**

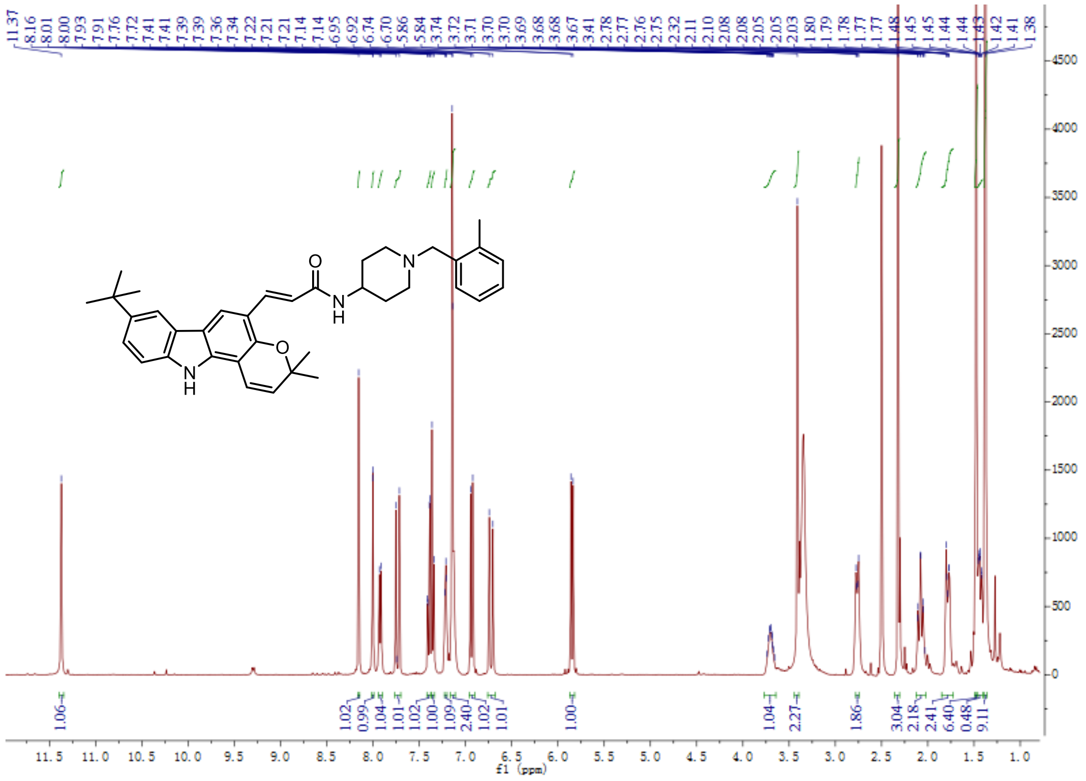

^{1}H NMR (DMSO, 400 MHz) Spectrum of Compound **6ac**

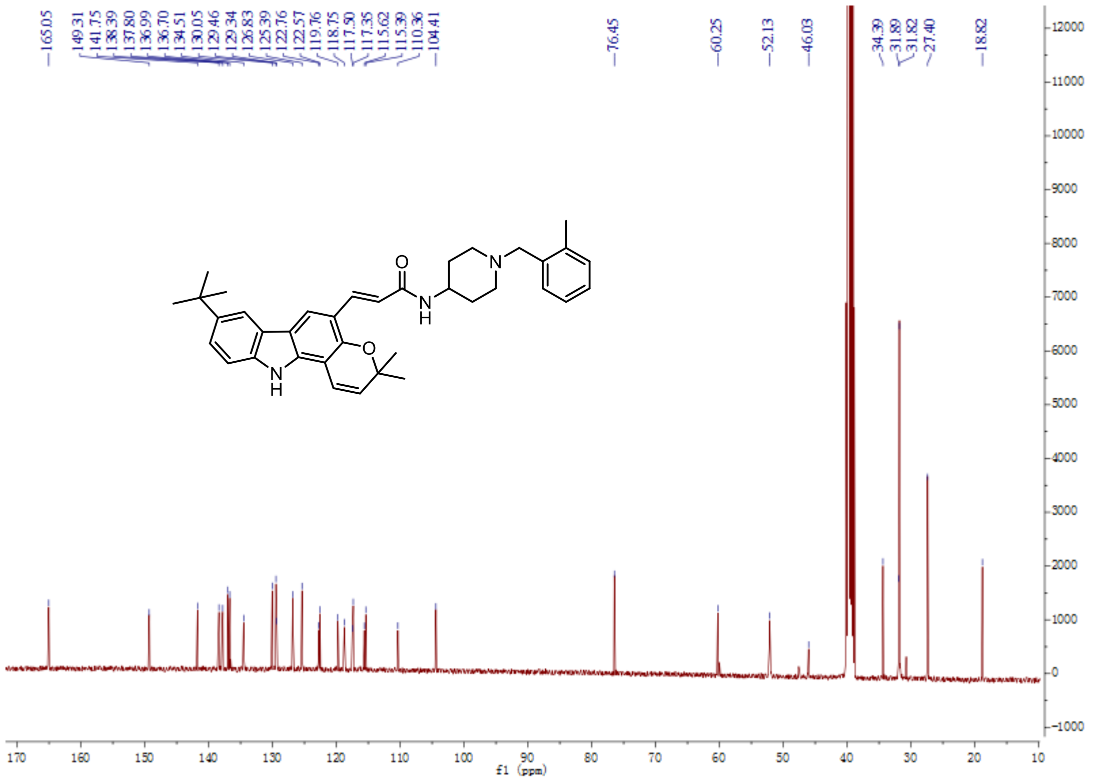

^{13}C NMR (DMSO, 400 MHz) Spectrum of Compound **6ac**

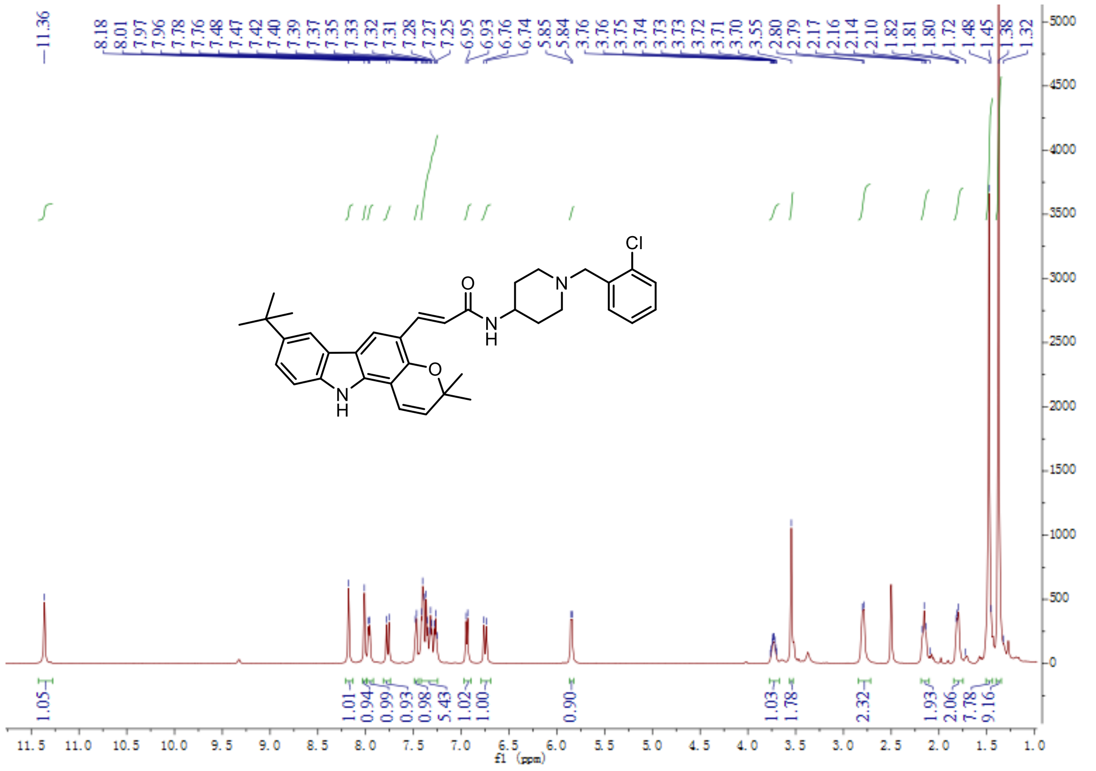

^{1}H NMR (DMSO, 400 MHz) Spectrum of Compound **6ad**

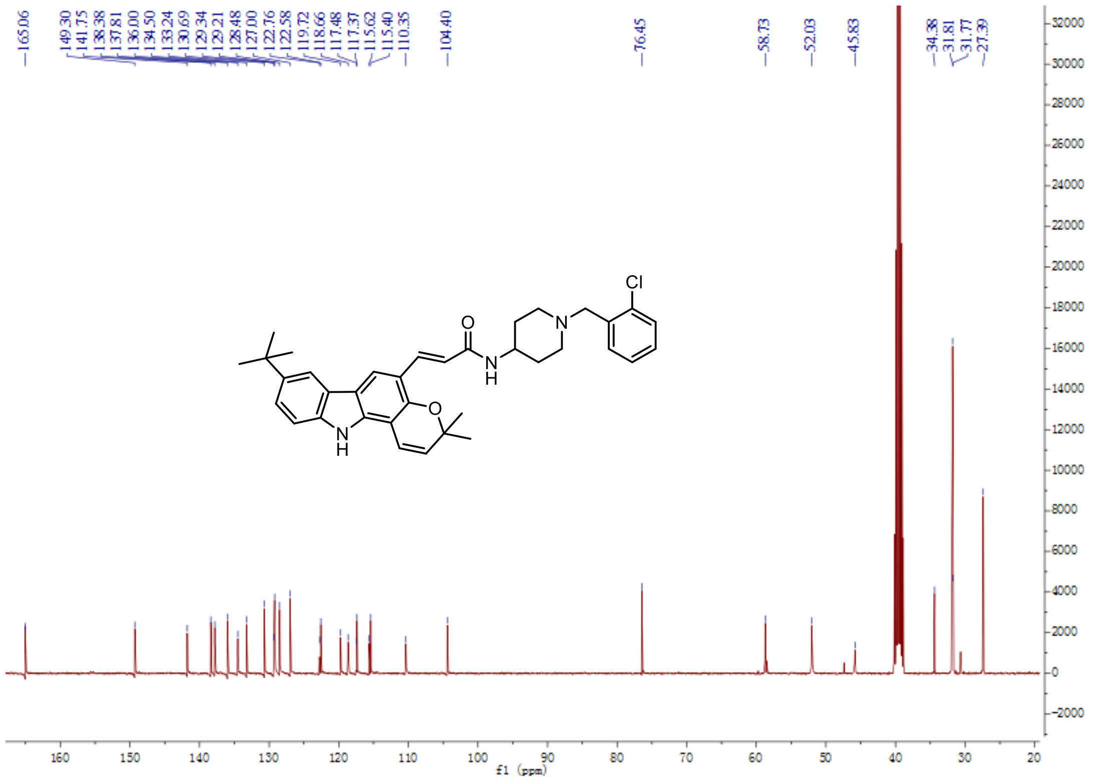

^{13}C NMR (DMSO, 400 MHz) Spectrum of Compound **6ad**

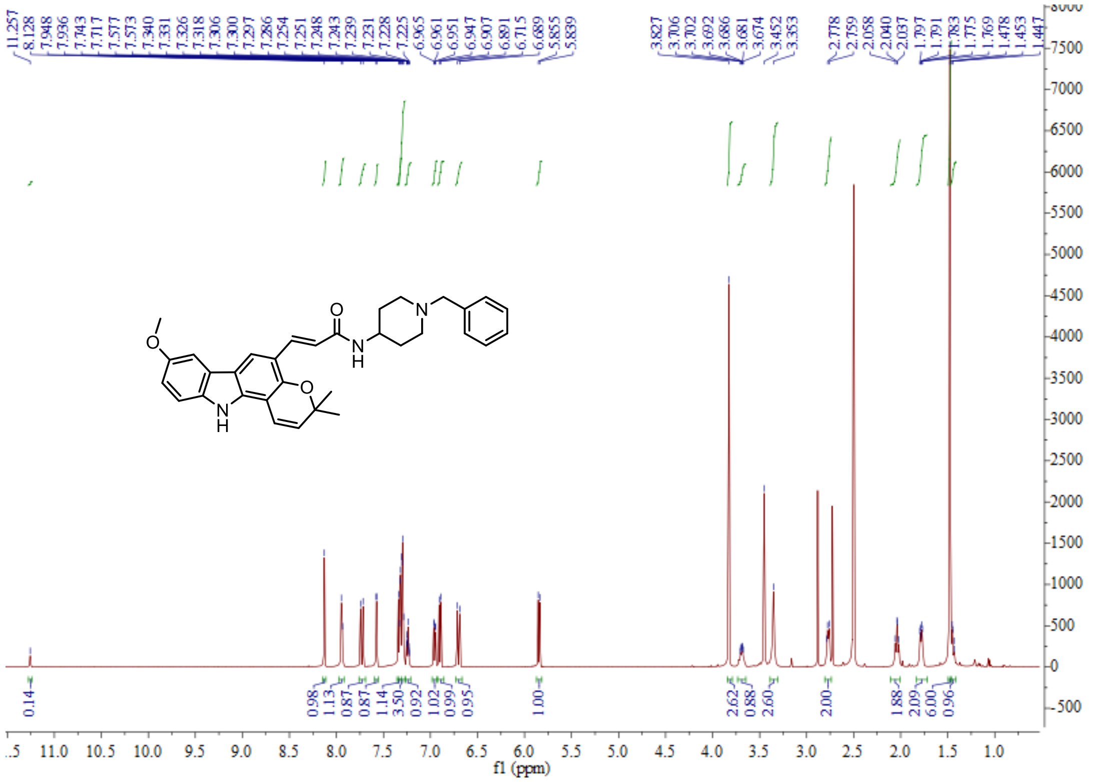

^{1}H NMR (DMSO, 600 MHz) Spectrum of Compound **6ae**

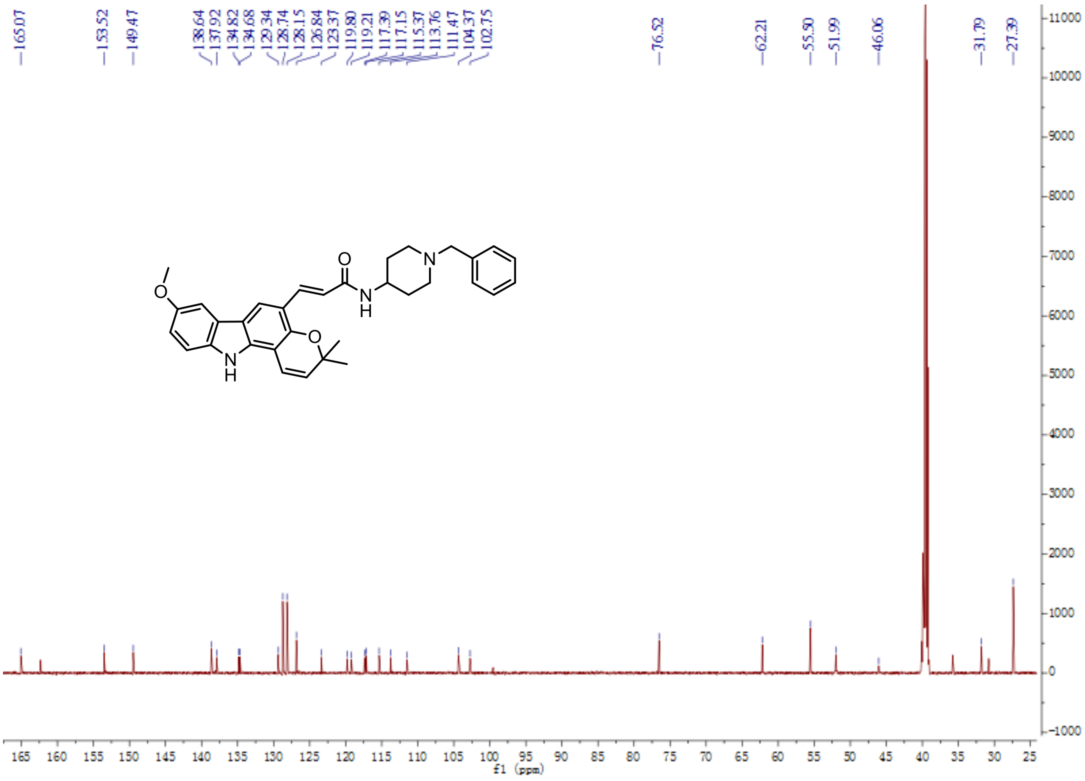

^{13}C NMR (DMSO, 600 MHz) Spectrum of Compound **6ae**

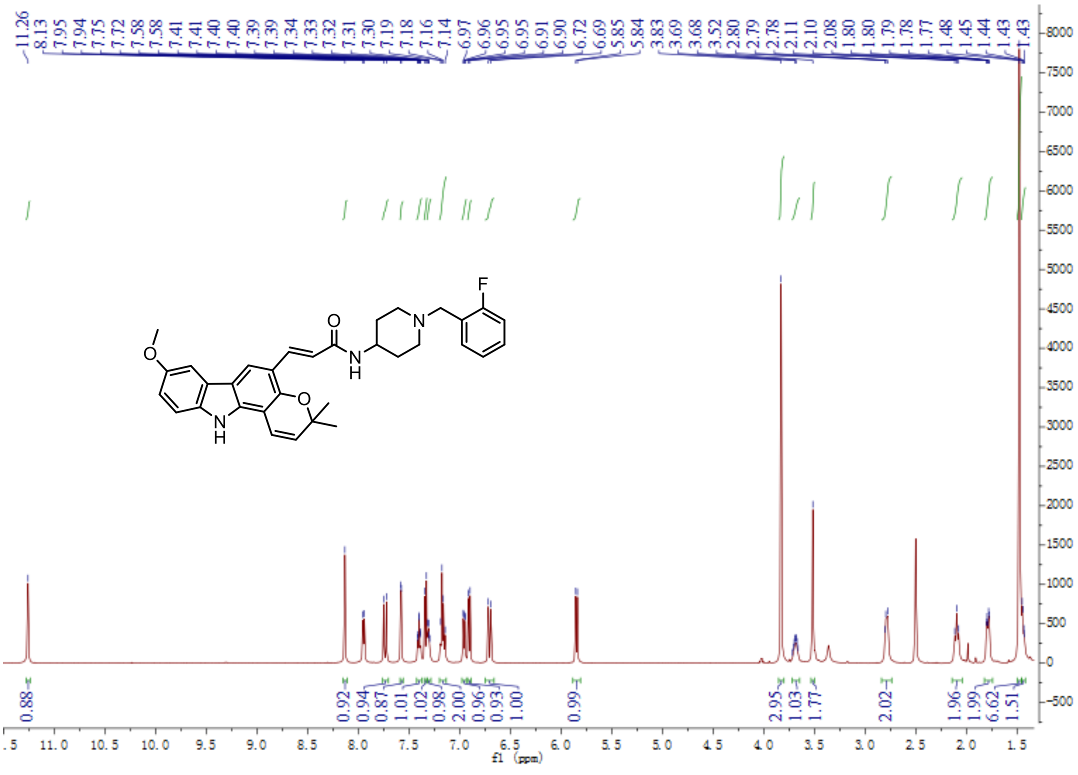

^{1}H NMR (DMSO, 600 MHz) Spectrum of Compound **6af**

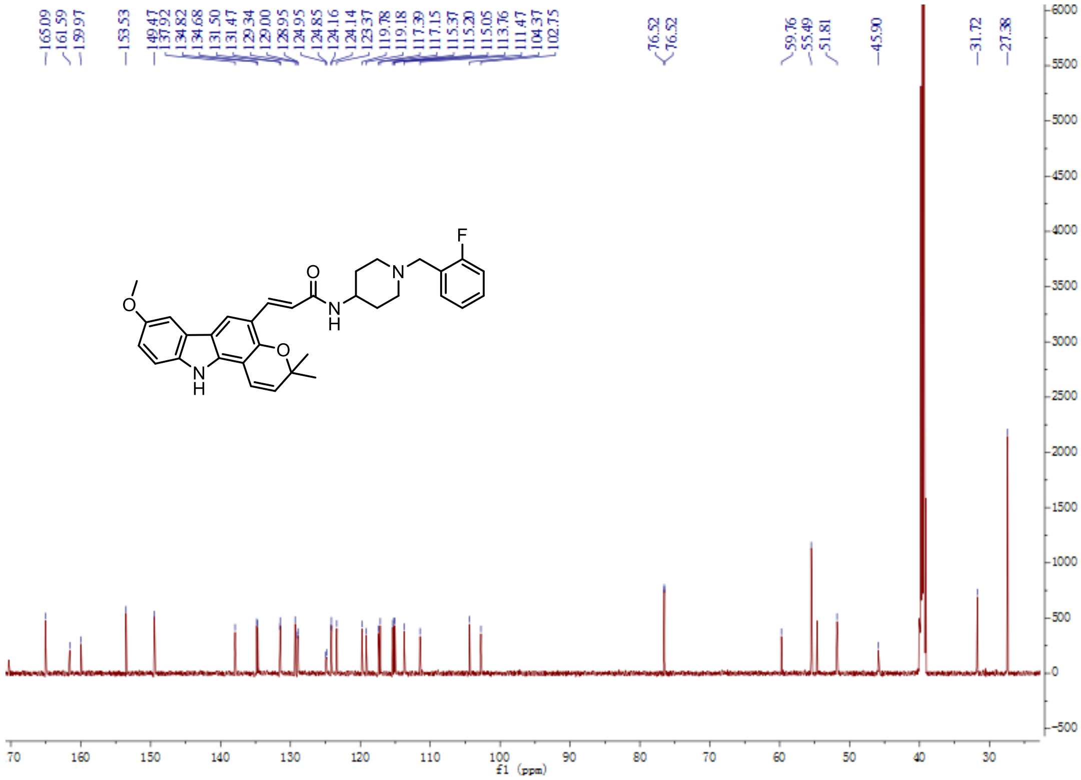

^{13}C NMR (DMSO, 600 MHz) Spectrum of Compound **6af**

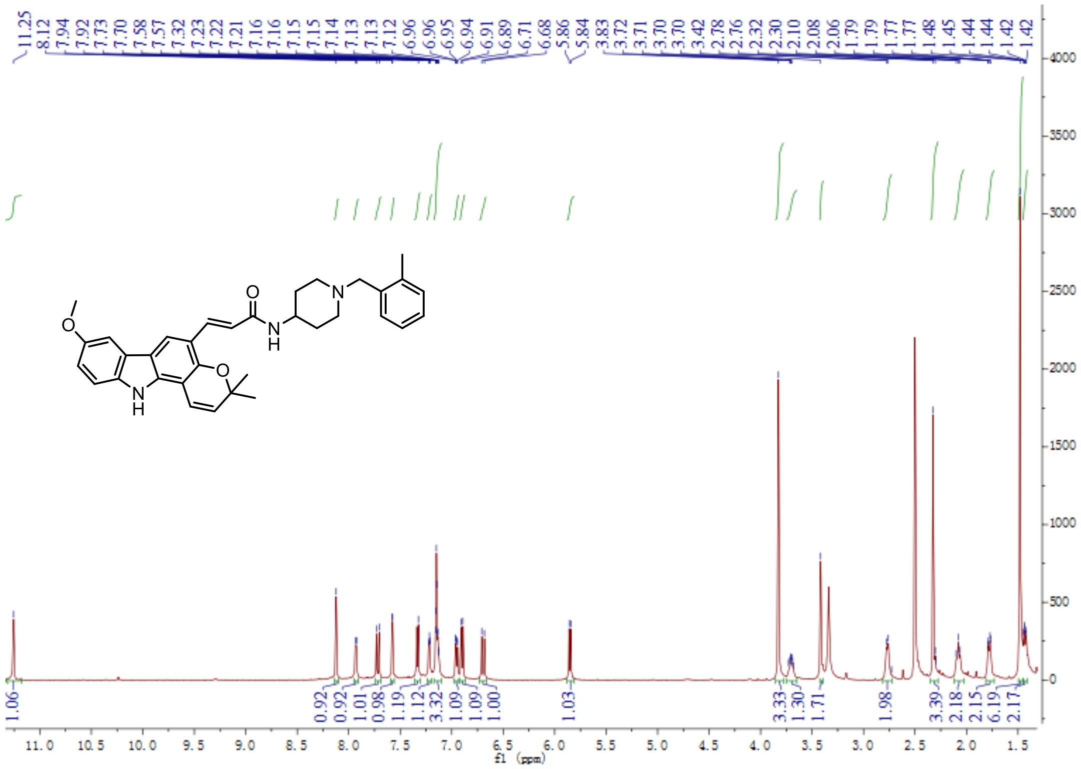

^{1}H NMR (DMSO, 600 MHz) Spectrum of Compound **6ag**

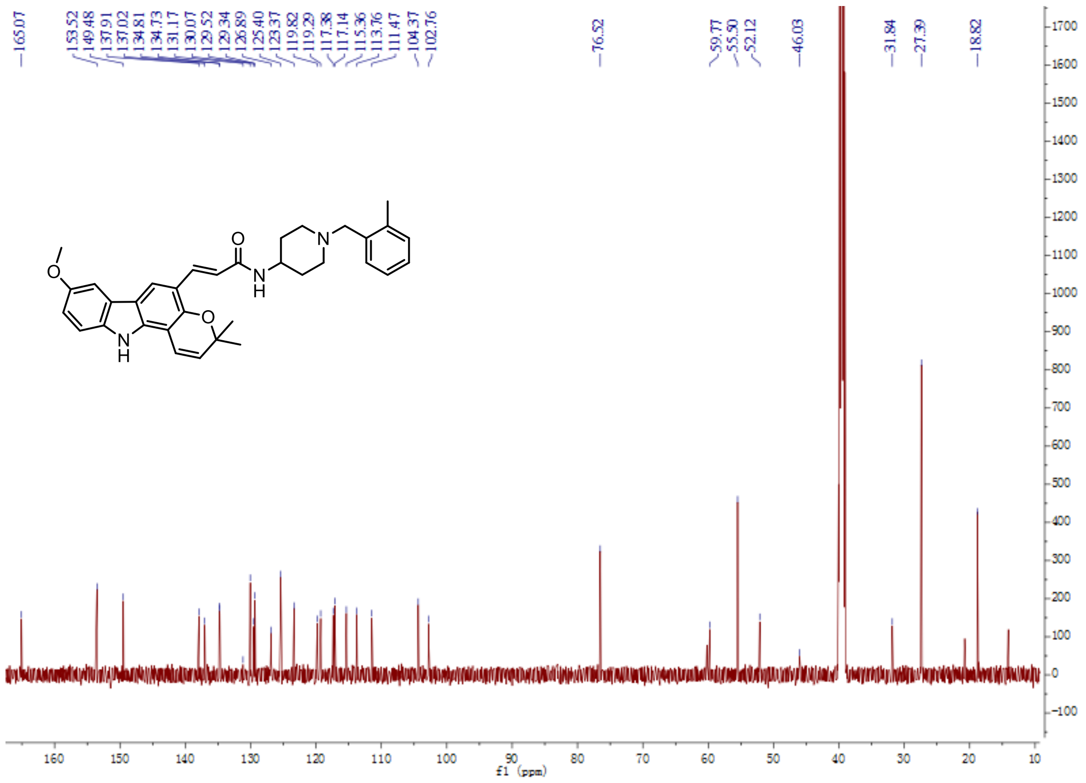

[13] C NMR (DMSO, 600 MHz) Spectrum of Compound **6ag**

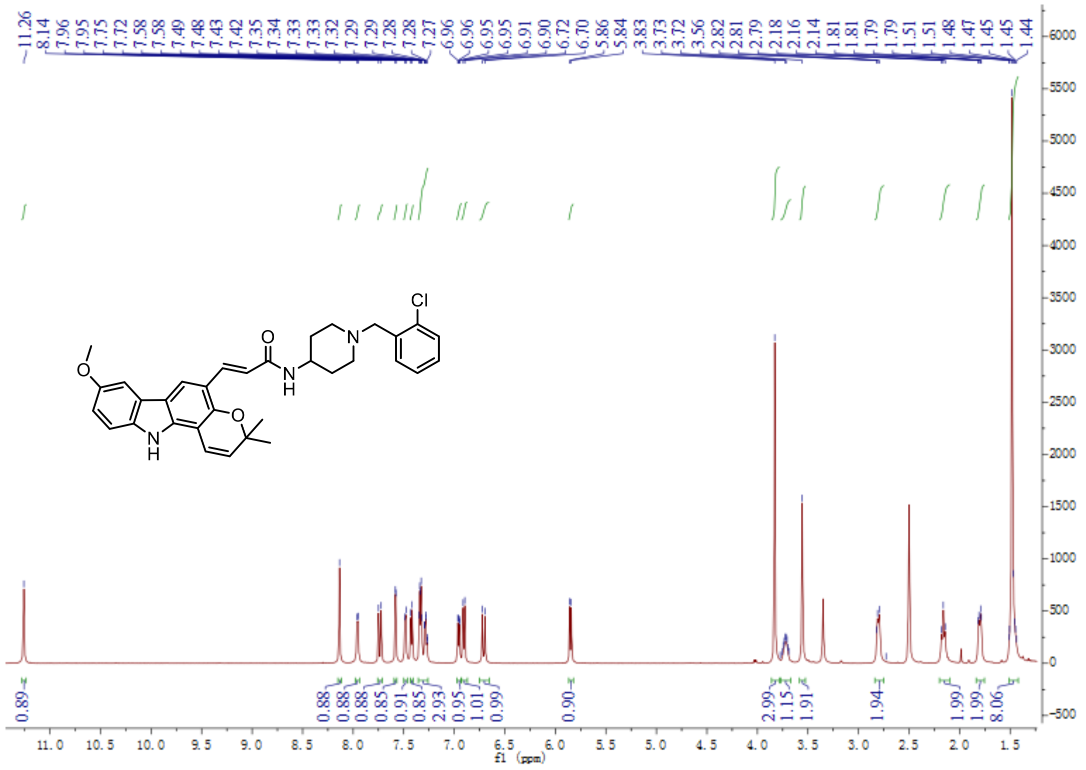

[1] H NMR (DMSO, 600 MHz) Spectrum of Compound **6ah**

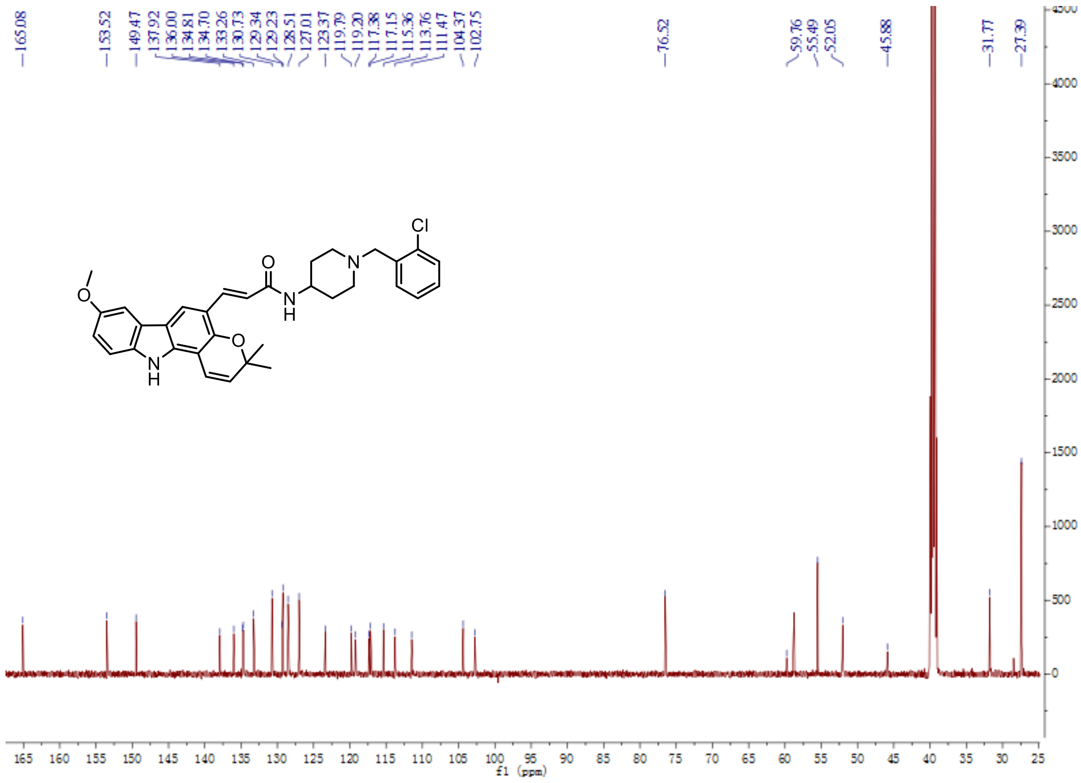

[13] C NMR (DMSO, 600 MHz) Spectrum of Compound **6ah**

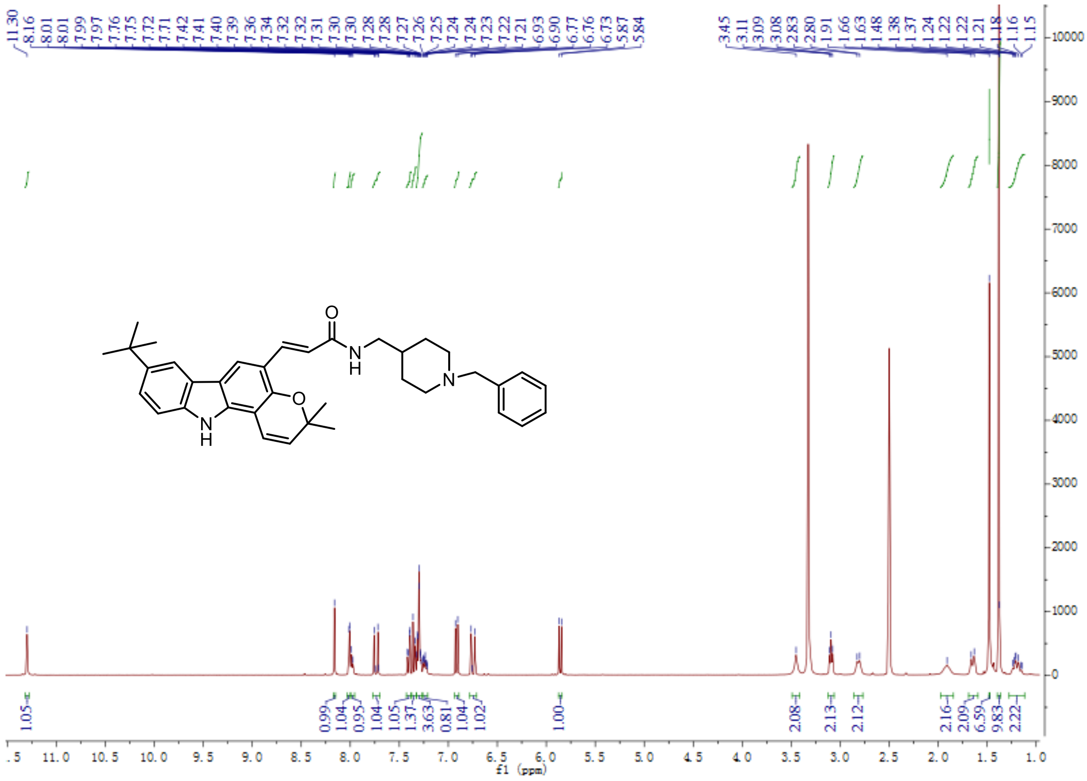

[1] H NMR (DMSO, 400 MHz) Spectrum of Compound **6ba**

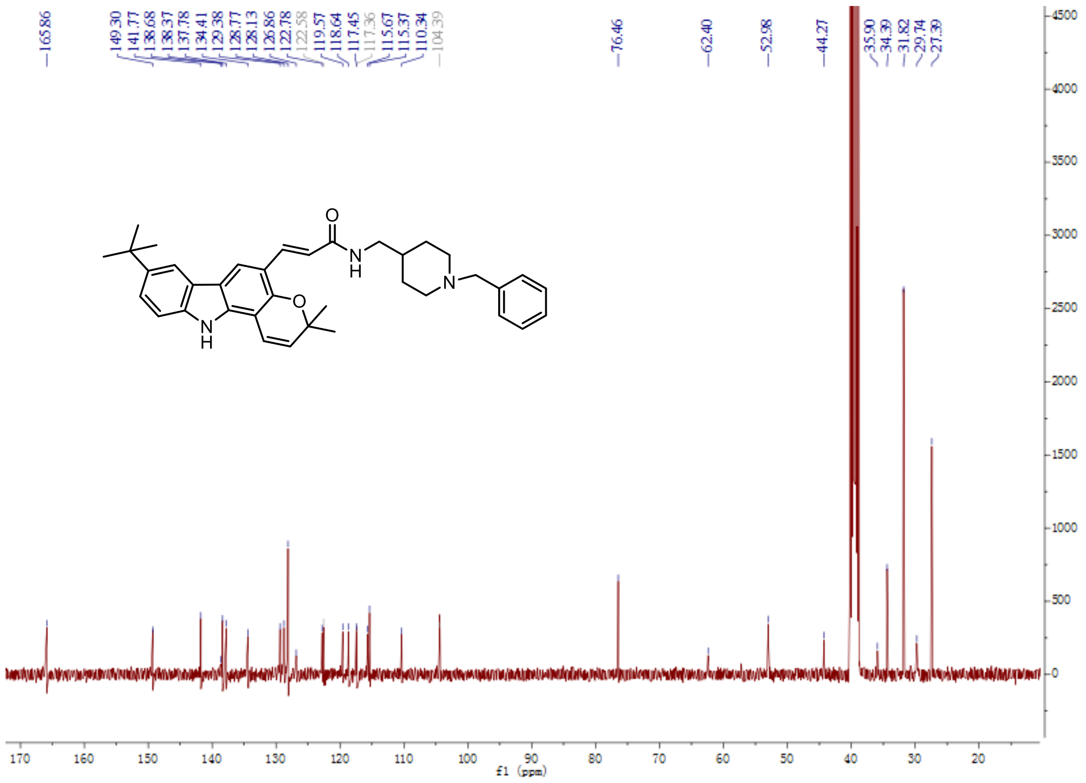

^{13}C NMR (DMSO, 400 MHz) Spectrum of Compound **6ba**

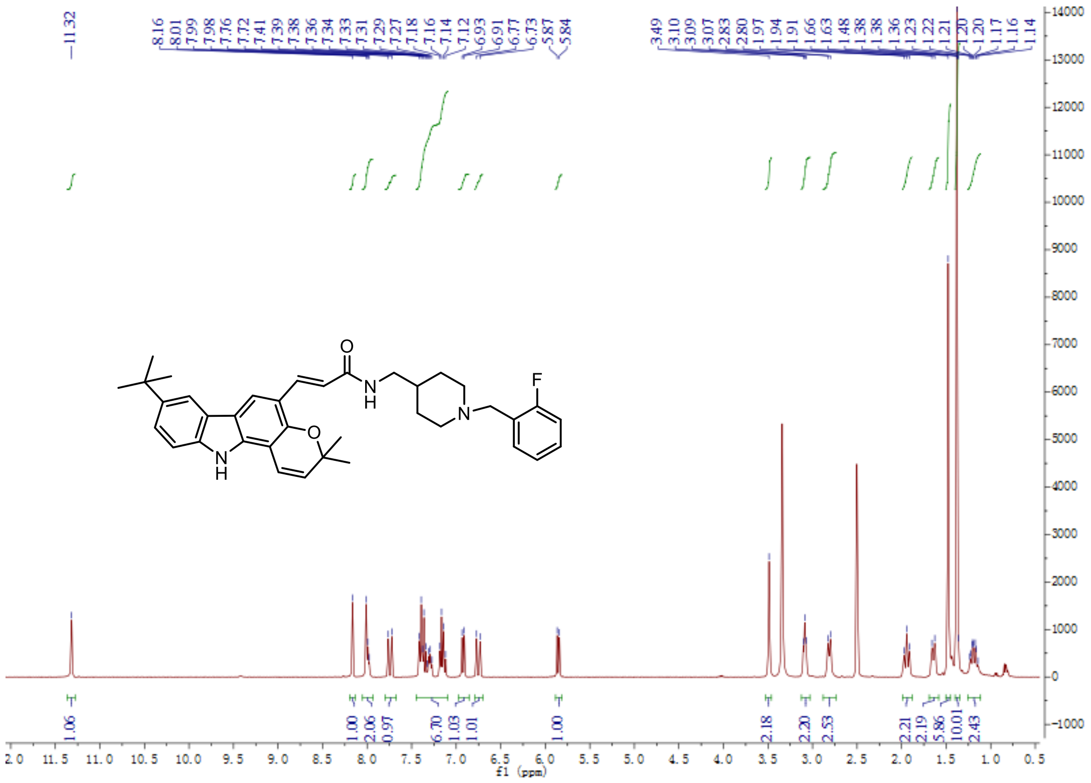

^{1}H NMR (DMSO, 400 MHz) Spectrum of Compound **6bb**

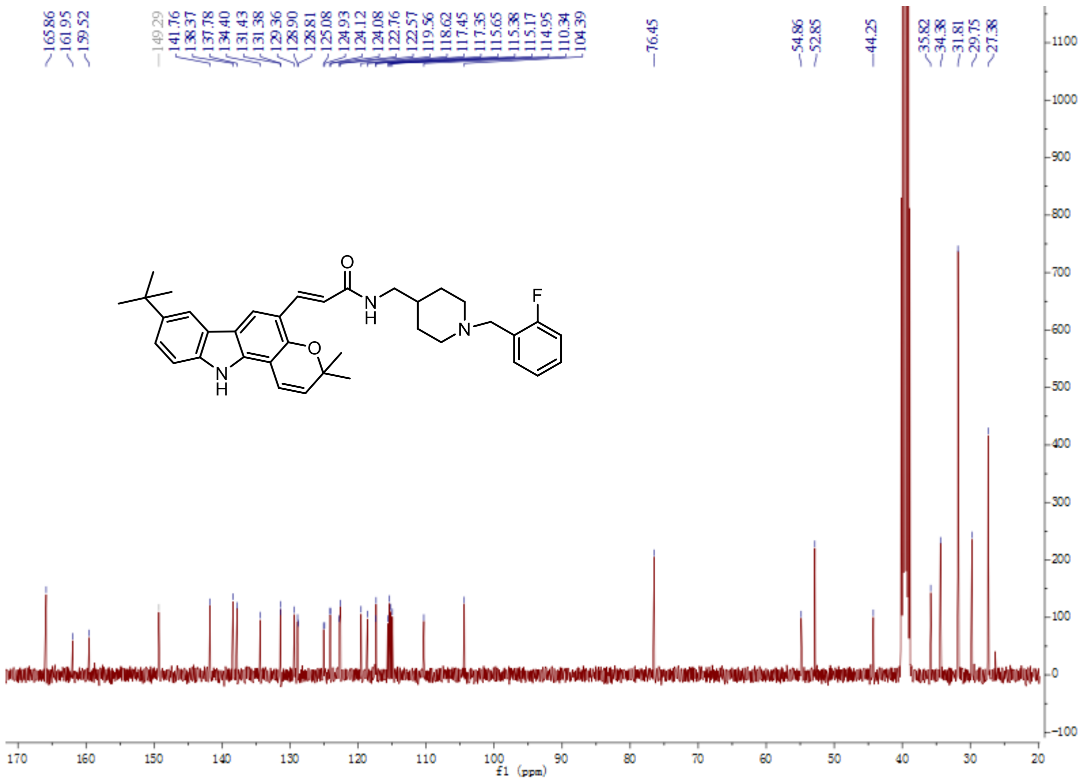

^{13}C NMR (DMSO, 400 MHz) Spectrum of Compound **6bb**

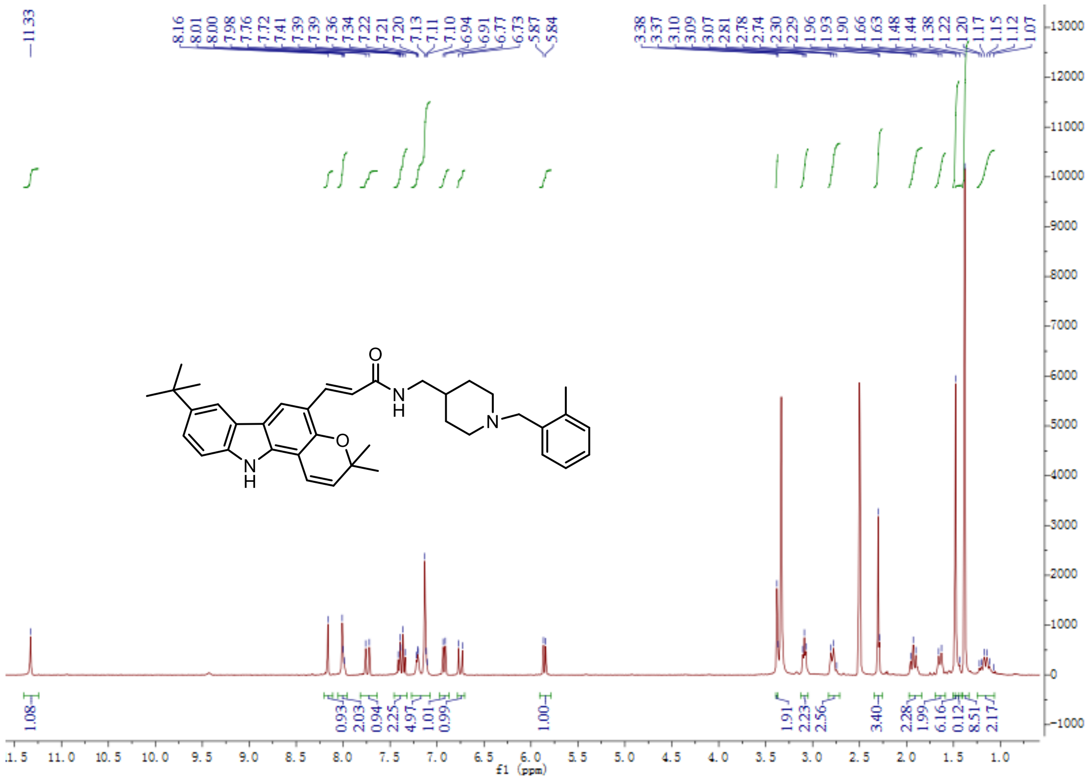

^{1}H NMR (DMSO, 400 MHz) Spectrum of Compound **6bc**

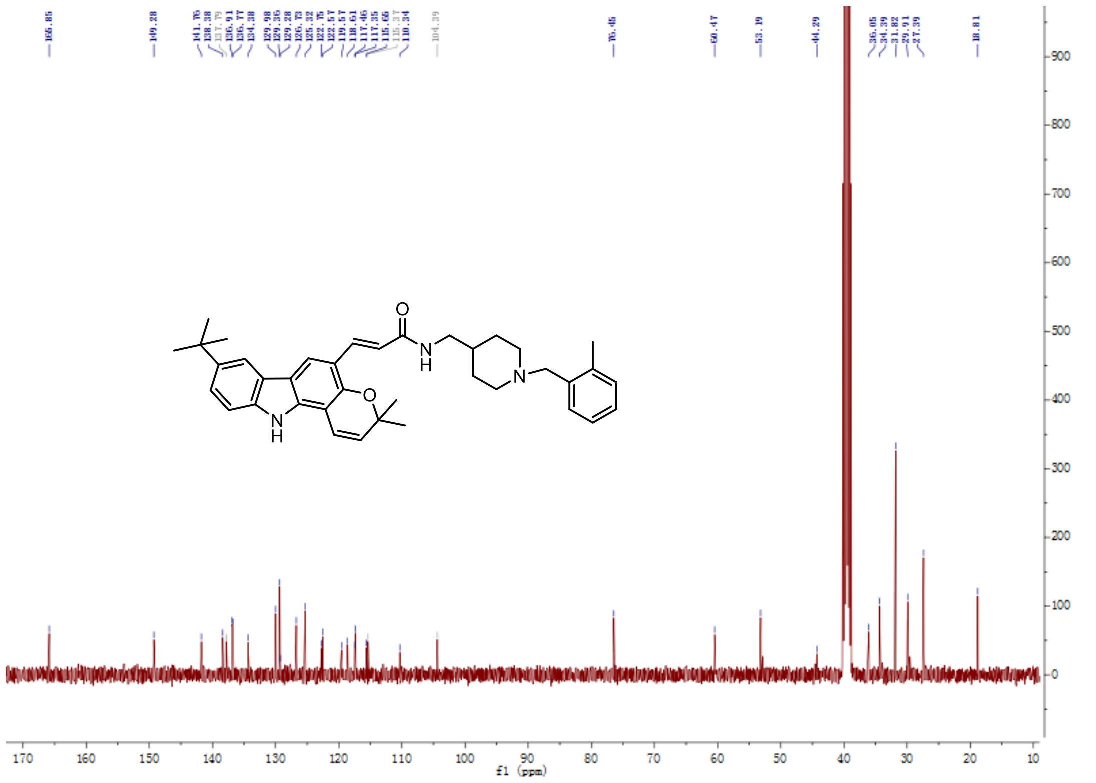

^{13}C NMR (DMSO, 400 MHz) Spectrum of Compound **6bc**

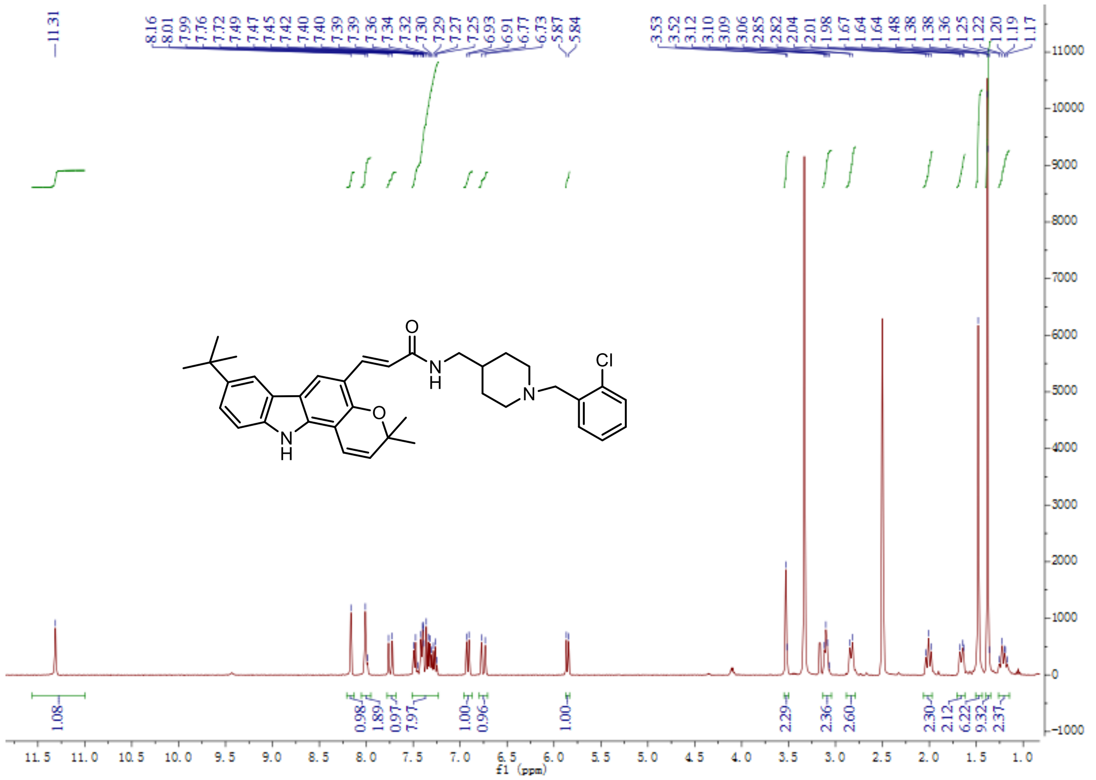

^{1}H NMR (DMSO, 400 MHz) Spectrum of Compound **6bd**

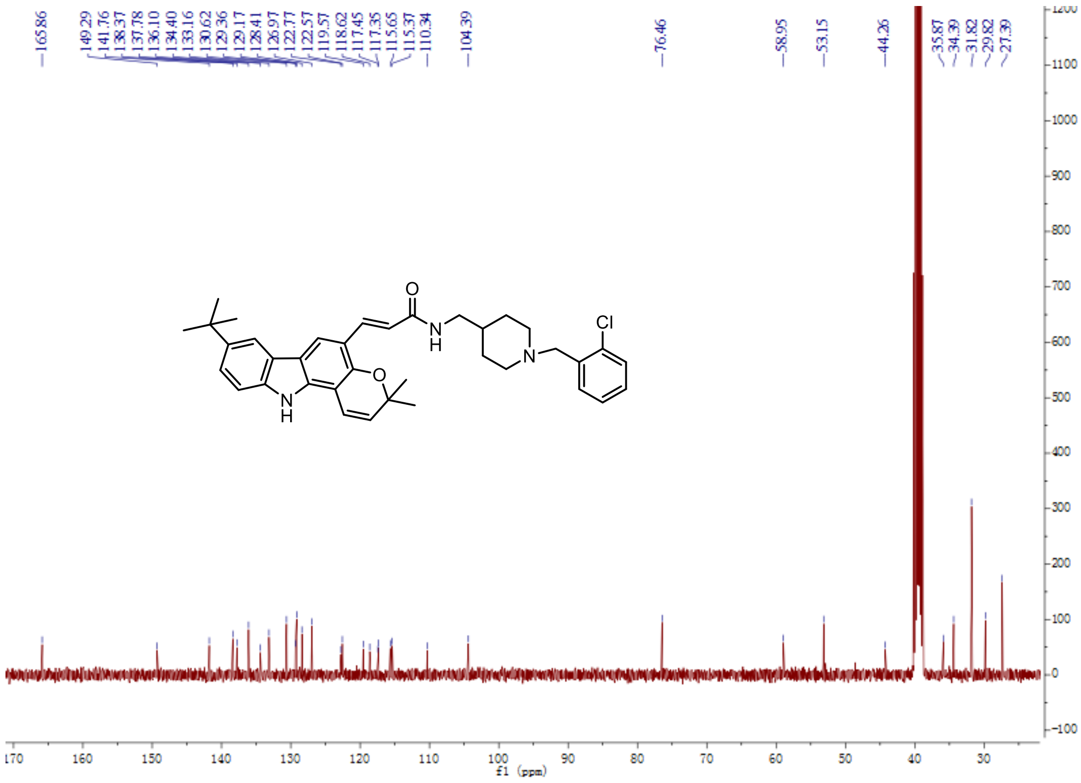

^{13} C NMR (DMSO, 400 MHz) Spectrum of Compound **6bd**

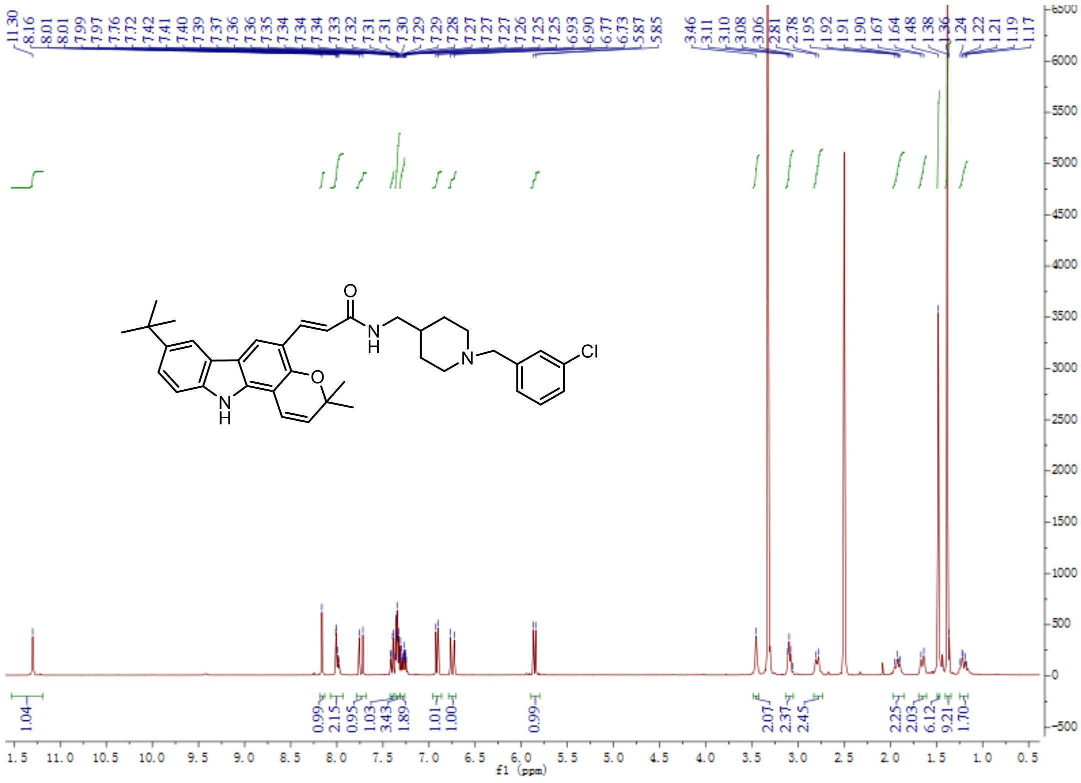

^{1} H NMR (DMSO, 400 MHz) Spectrum of Compound **6be**

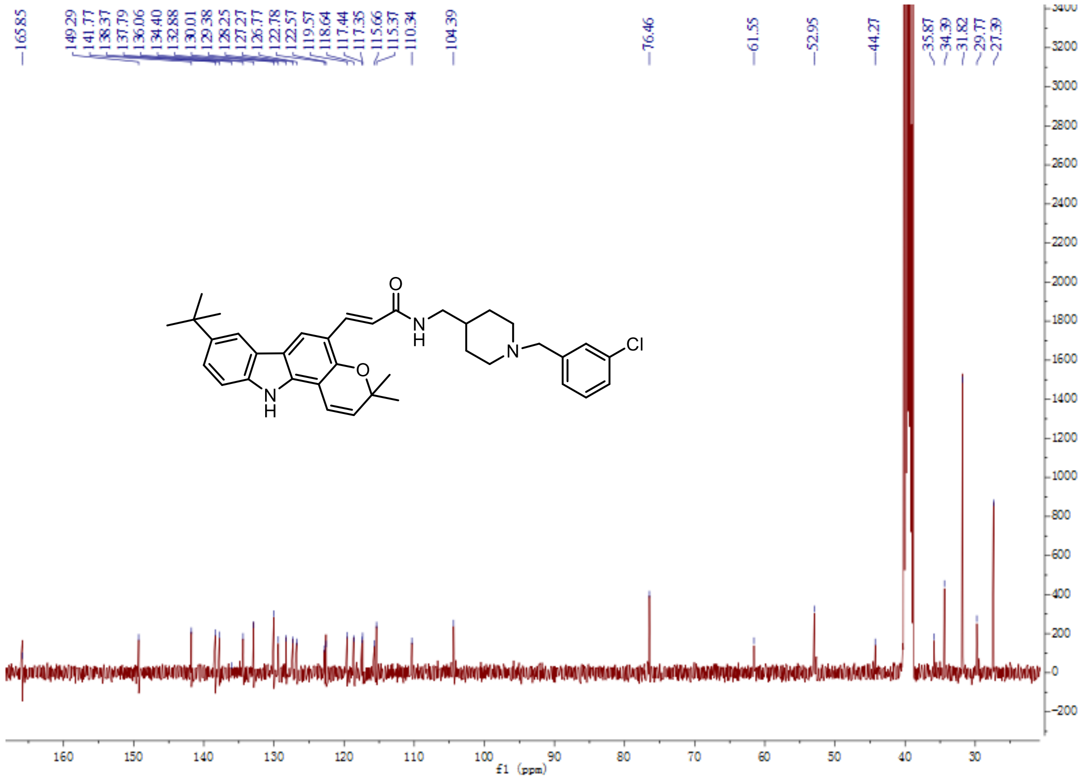

^{13} C NMR (DMSO, 400 MHz) Spectrum of Compound **6be**

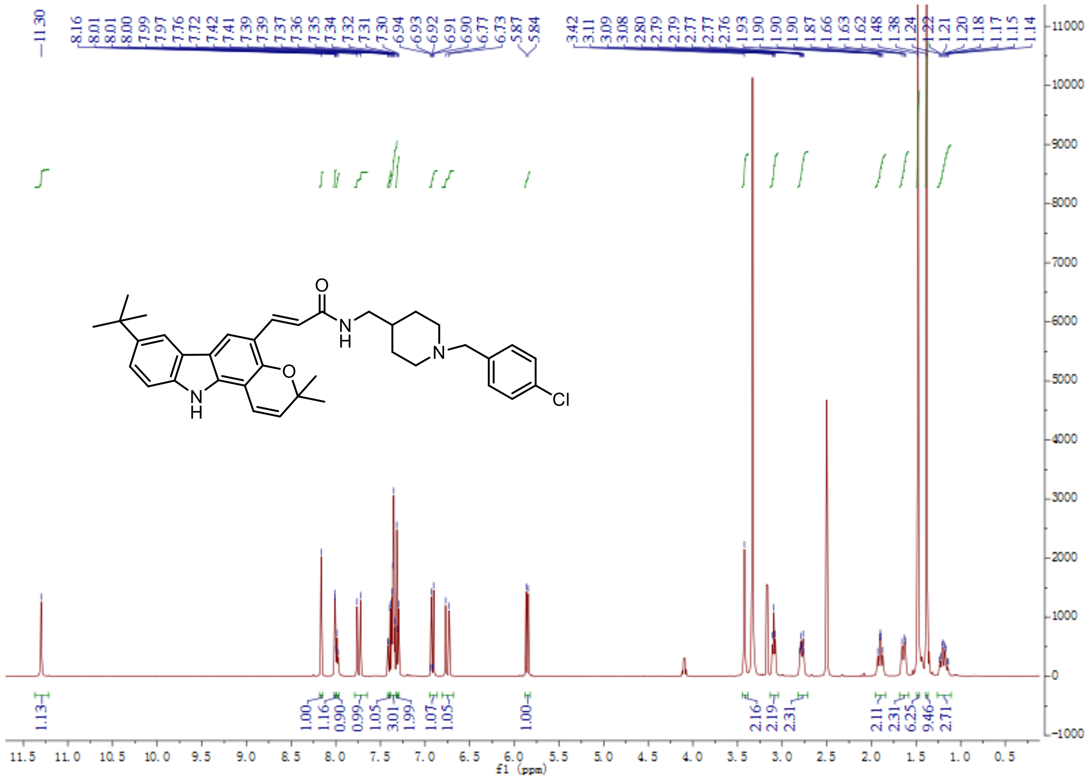

^{1} H NMR (DMSO, 400 MHz) Spectrum of Compound **6bf**

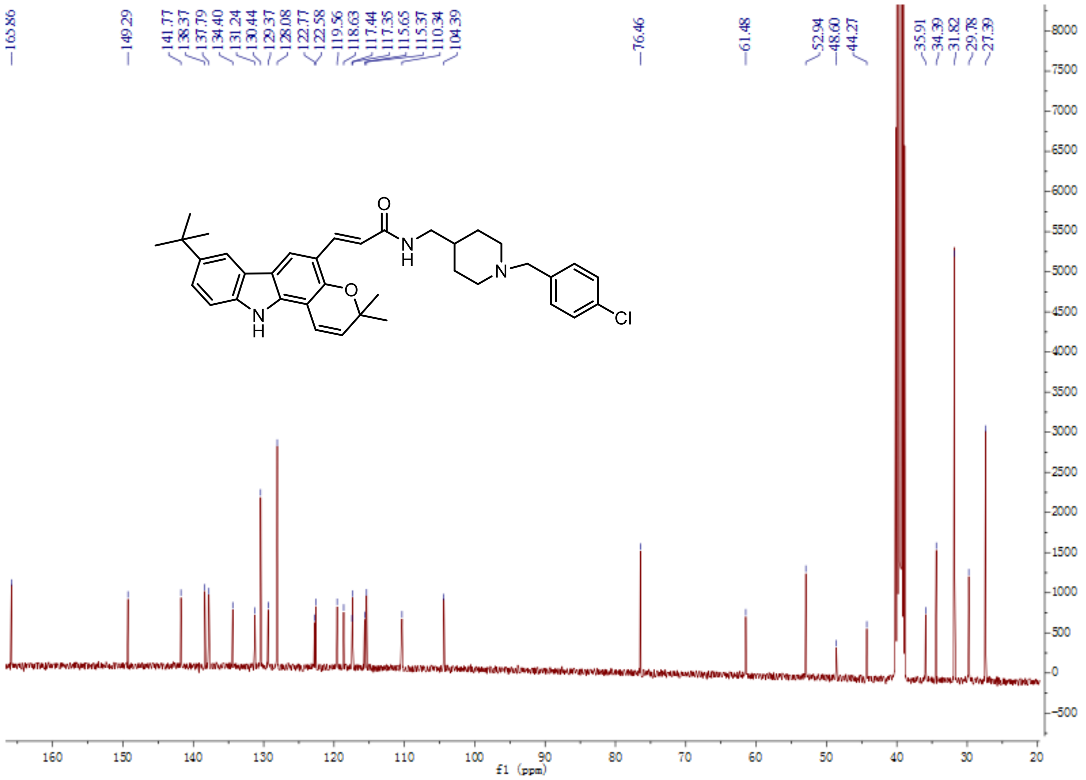

^{13} C NMR (DMSO, 400 MHz) Spectrum of Compound **6bf**

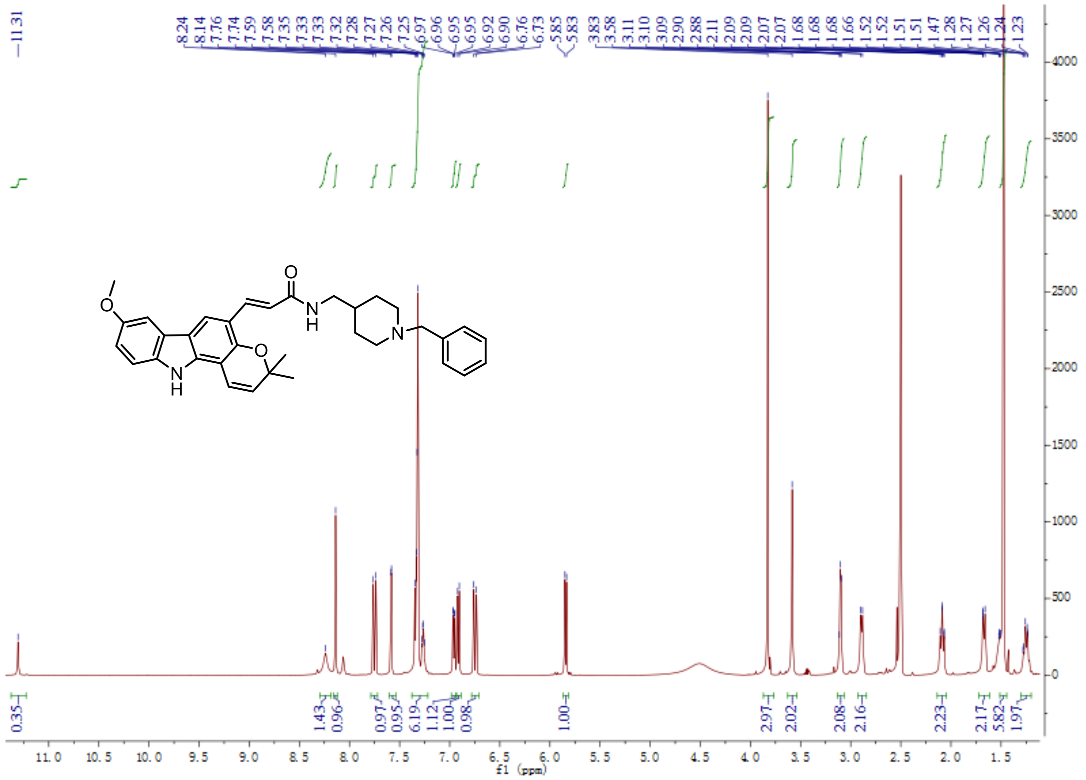

^{1} H NMR (DMSO, 600 MHz) Spectrum of Compound **6bg**

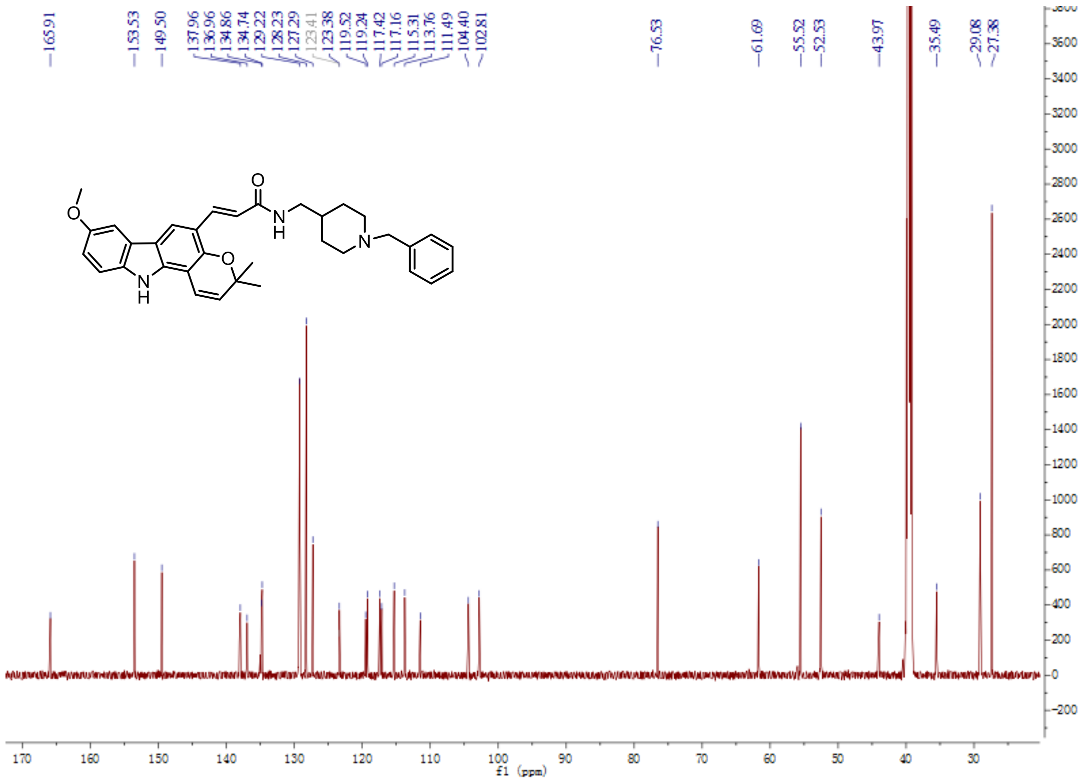

^{13}C NMR (DMSO, 600 MHz) Spectrum of Compound **6bg**

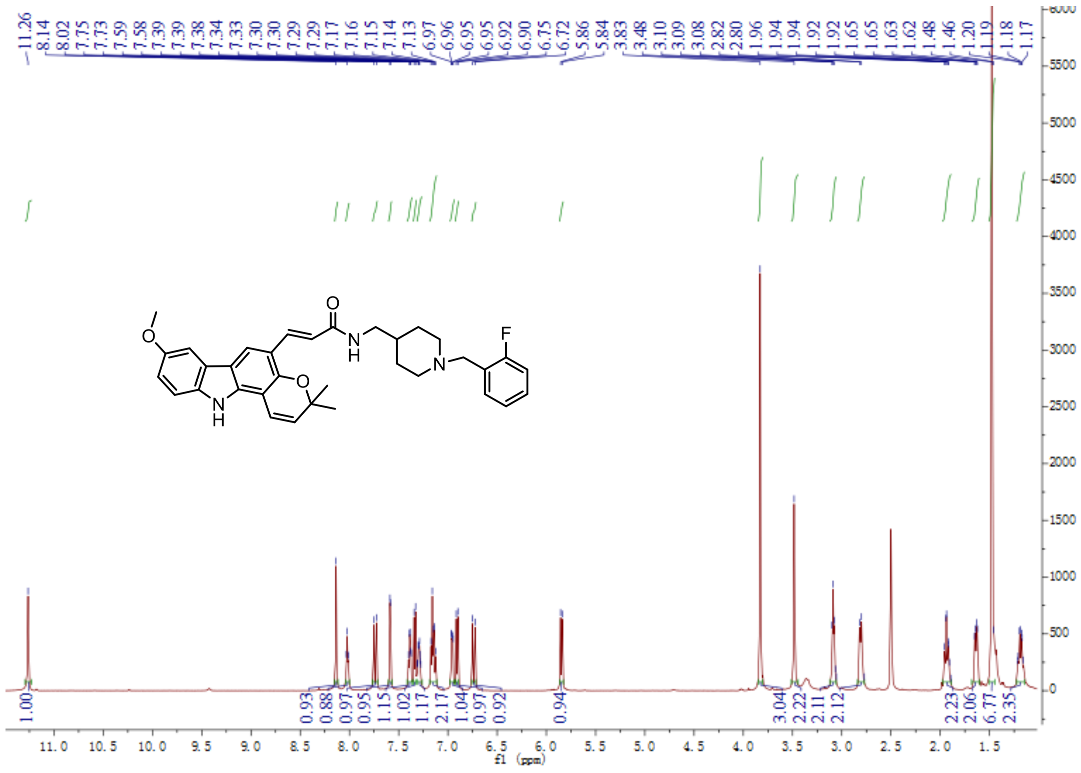

^{1}H NMR (DMSO, 600 MHz) Spectrum of Compound **6bh**

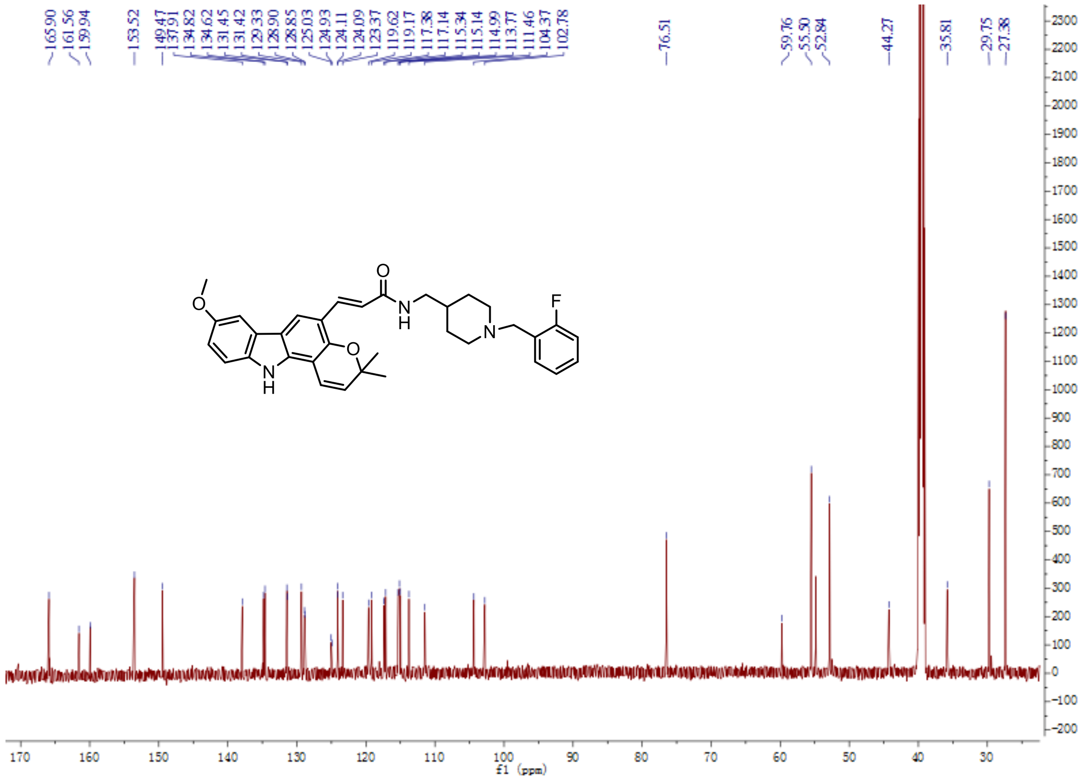

^{13} C NMR (DMSO, 600 MHz) Spectrum of Compound **6bh**

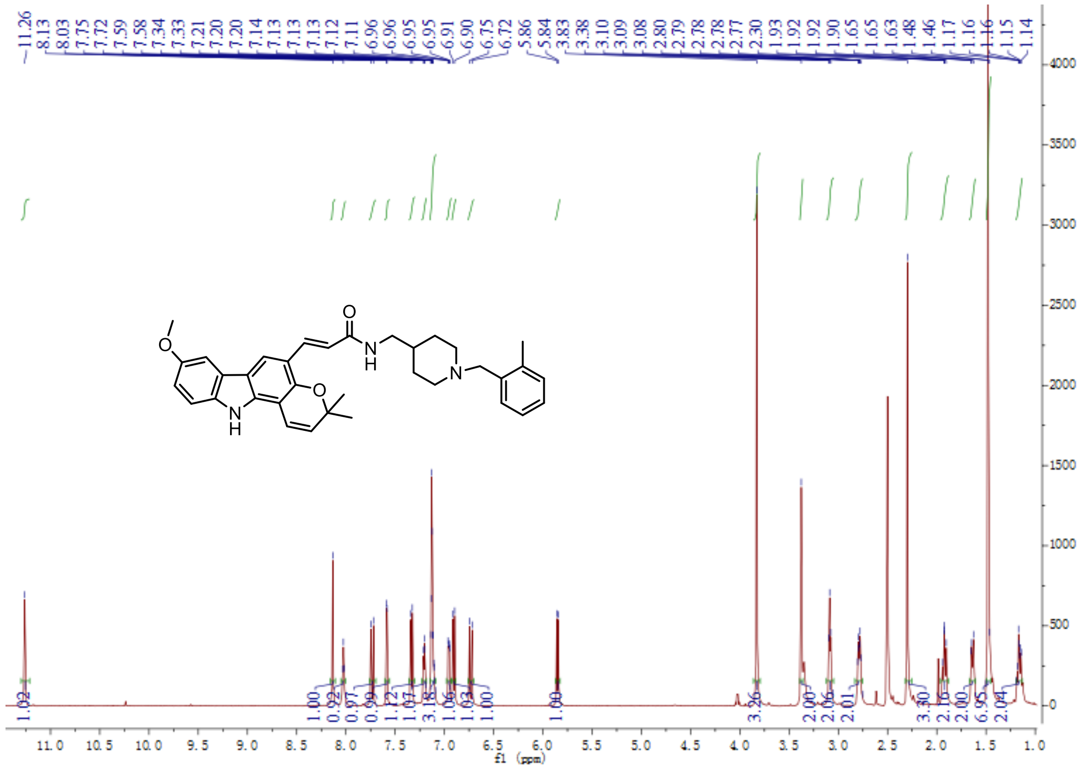

^{1} H NMR (DMSO, 600 MHz) Spectrum of Compound **6bi**

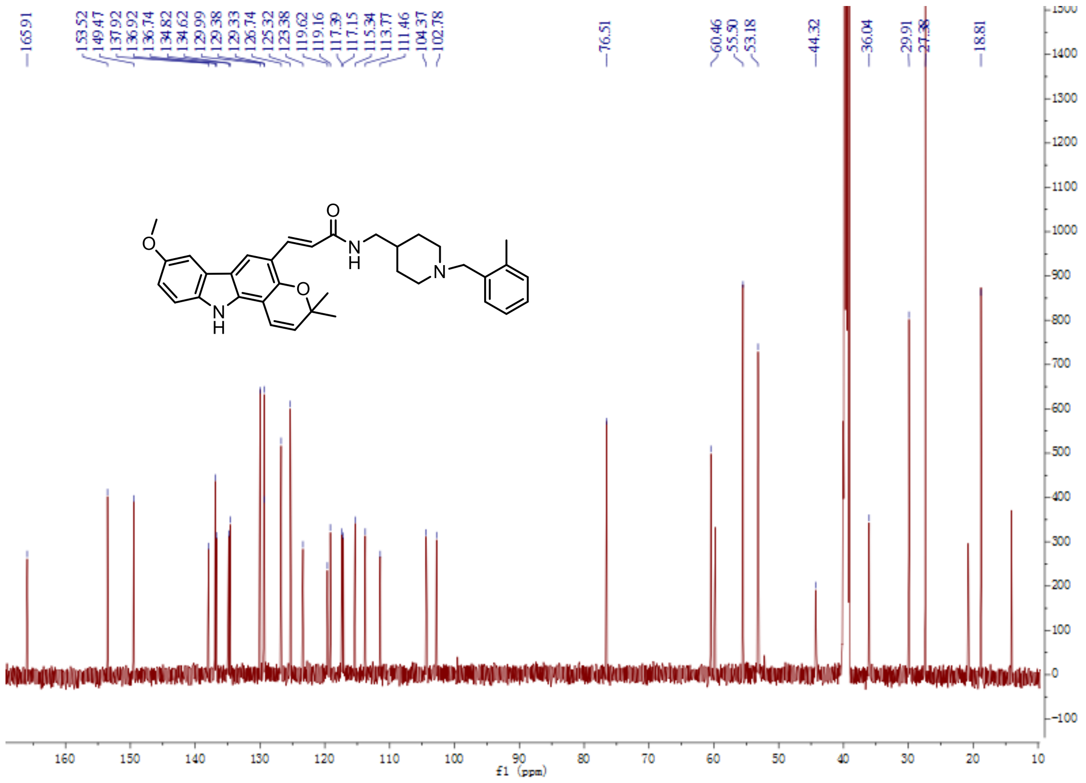

[13] C NMR (DMSO, 600 MHz) Spectrum of Compound **6bi**

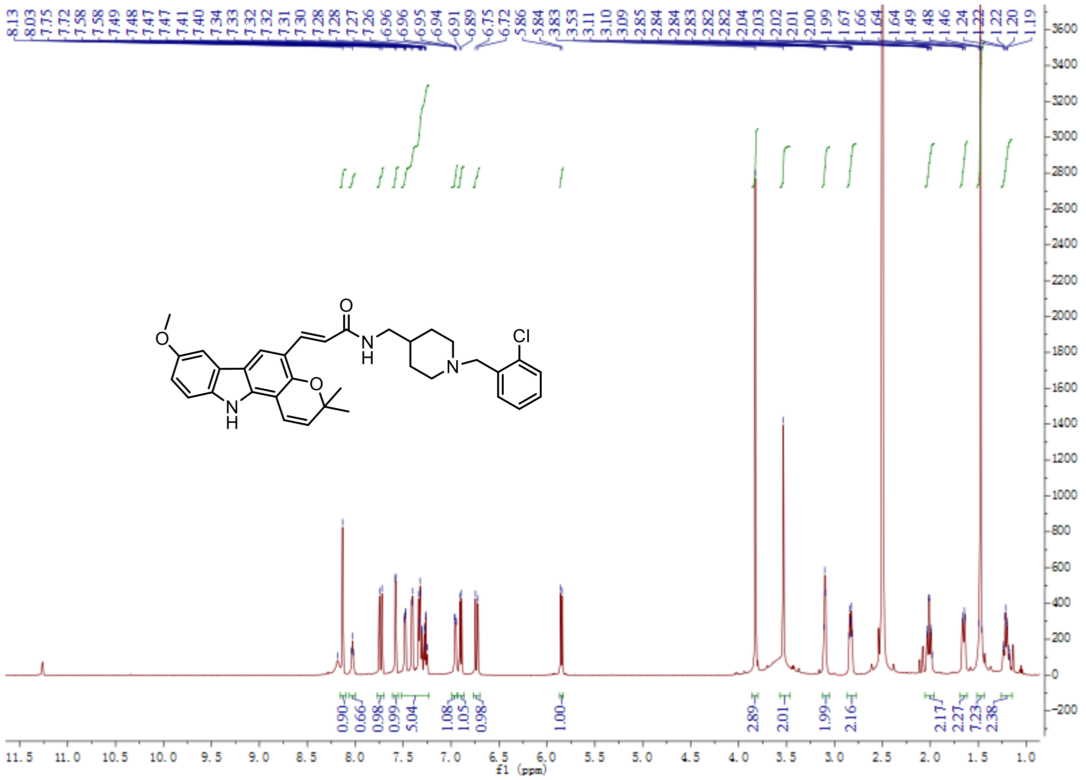

[1] H NMR (DMSO, 600 MHz) Spectrum of Compound **6bj**

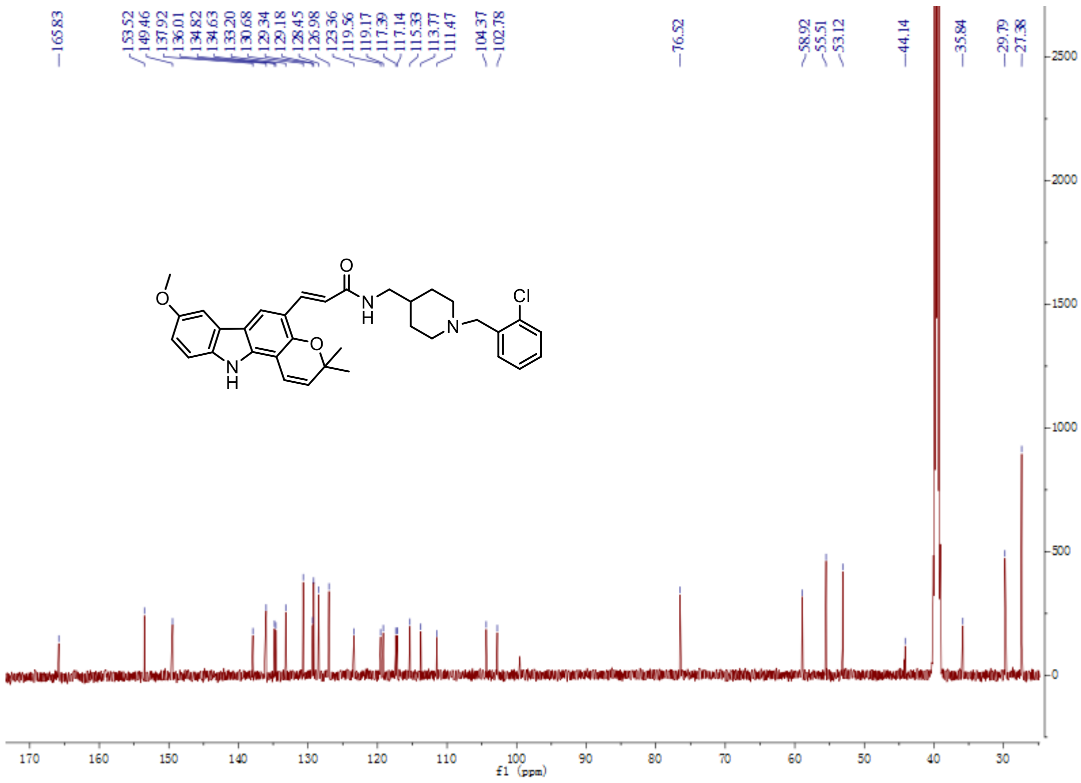

[13] C NMR (DMSO, 600 MHz) Spectrum of Compound **6bj**

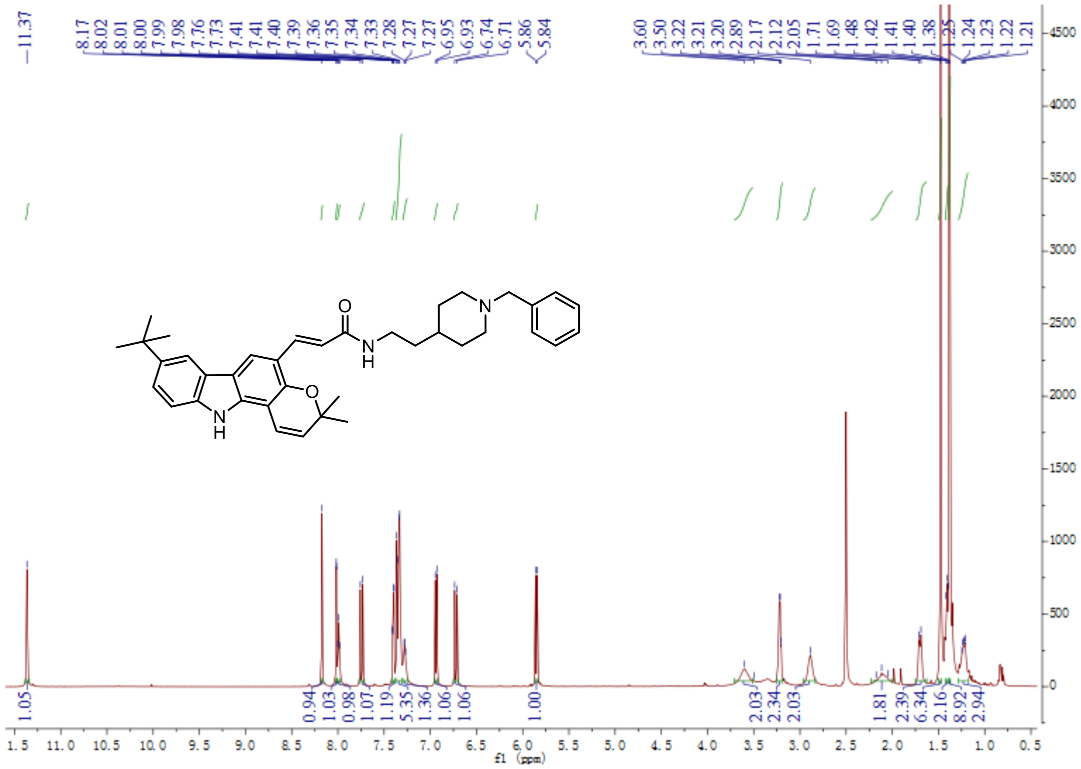

[1] H NMR (DMSO, 400 MHz) Spectrum of Compound **6ca**

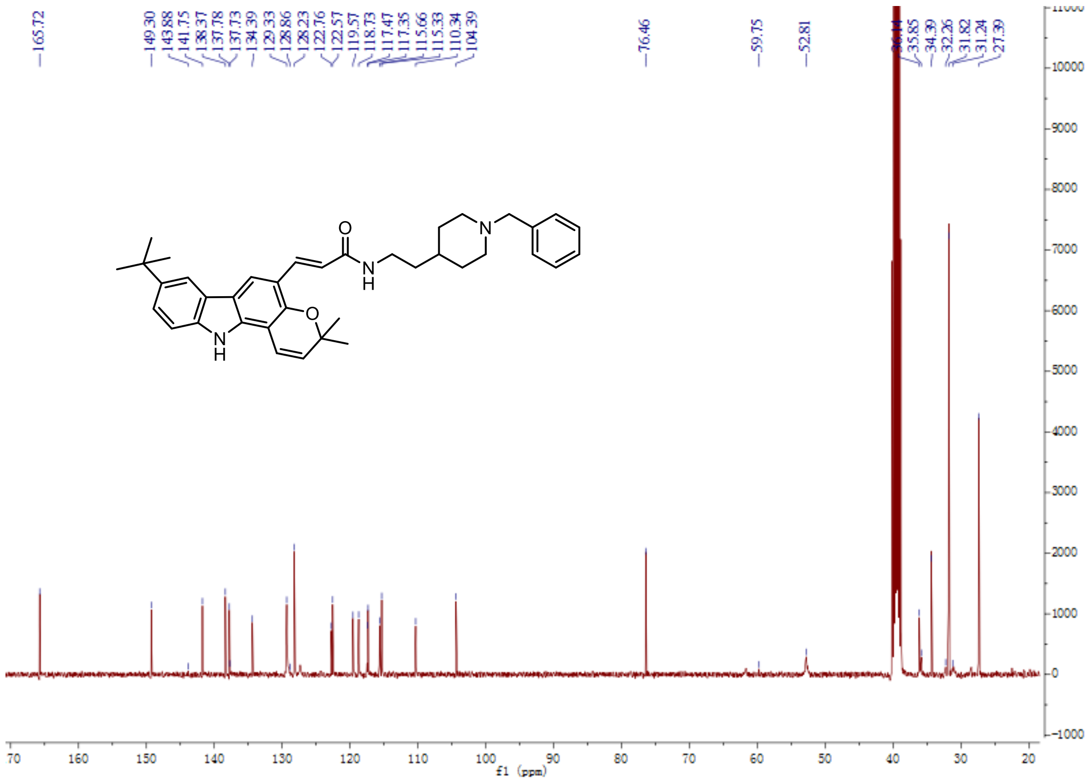

^{13}C NMR (DMSO, 400 MHz) Spectrum of Compound **6ca**

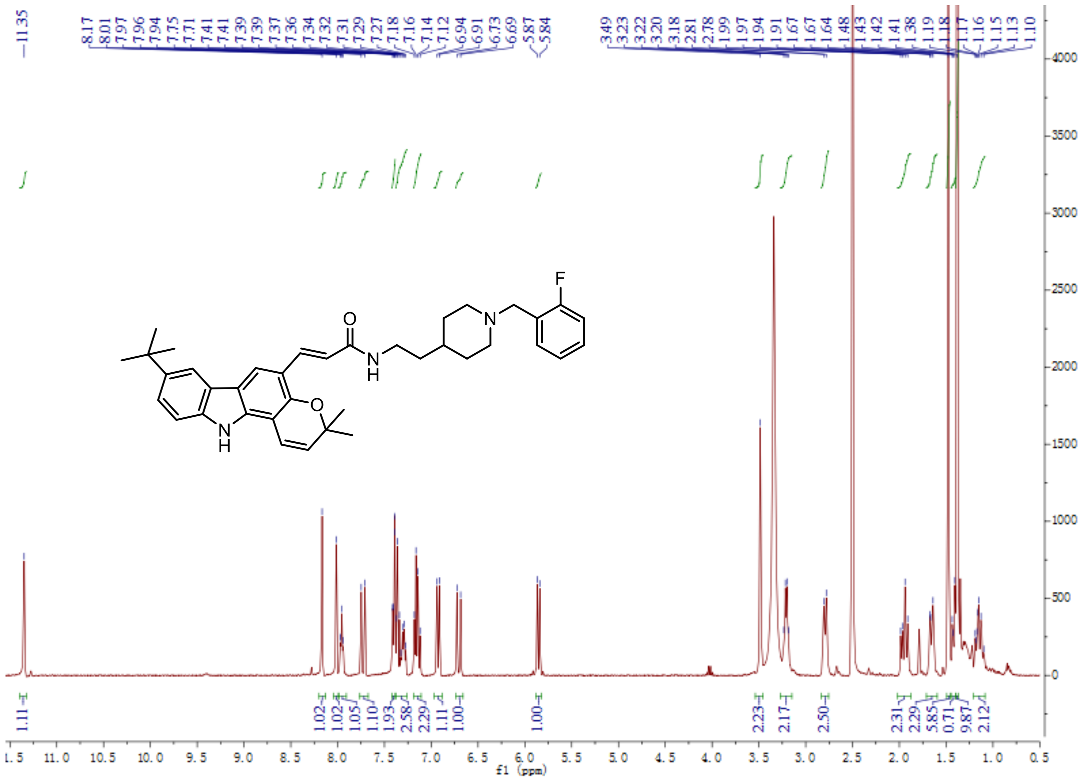

^{1}H NMR (DMSO, 400 MHz) Spectrum of Compound **6cb**

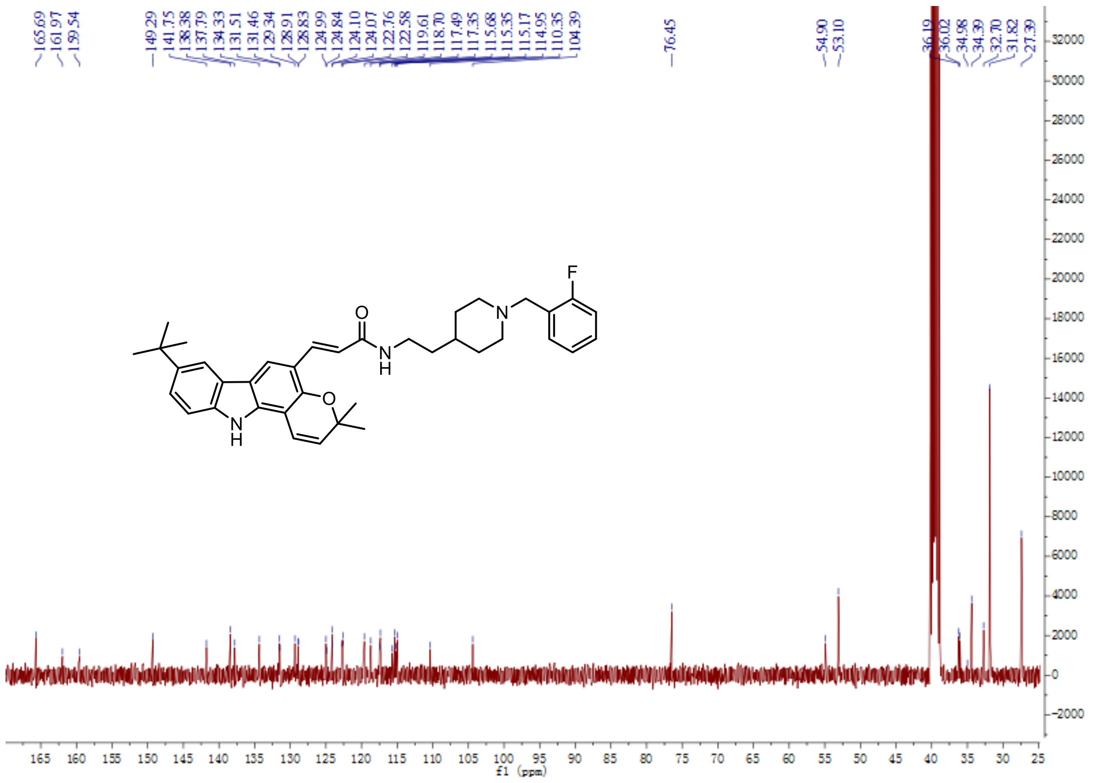

[13] C NMR (DMSO, 400 MHz) Spectrum of Compound **6cb**

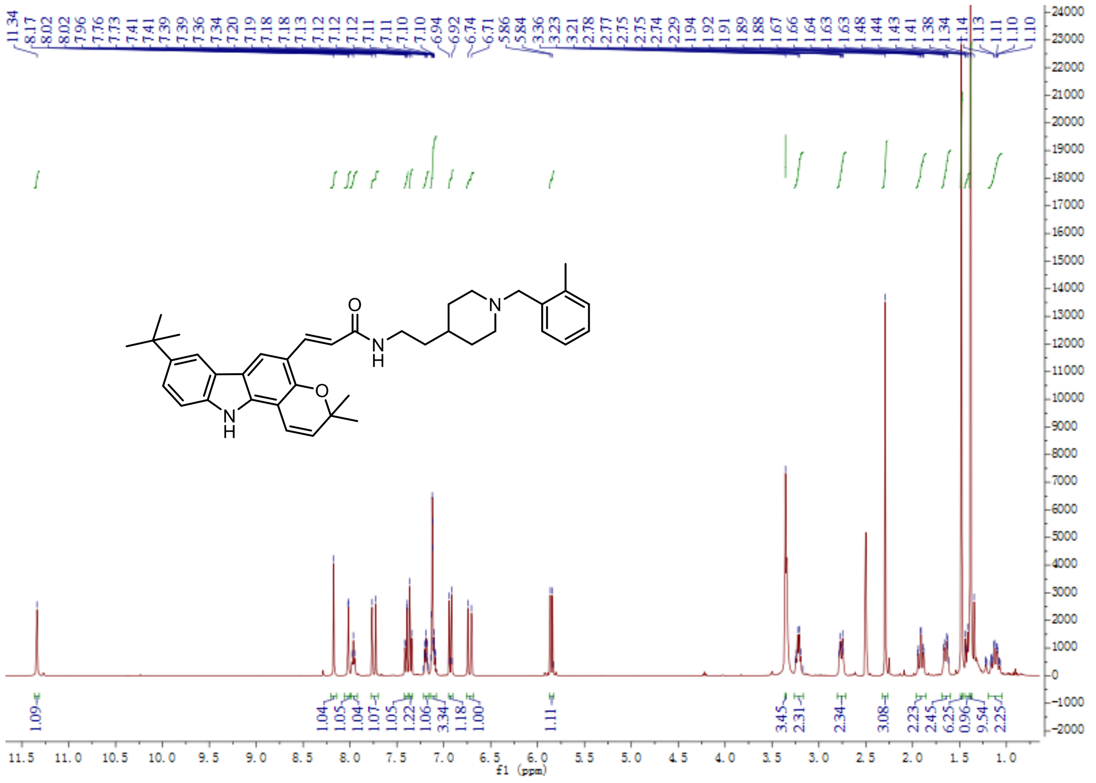

[1] H NMR (DMSO, 400 MHz) Spectrum of Compound **6cc**

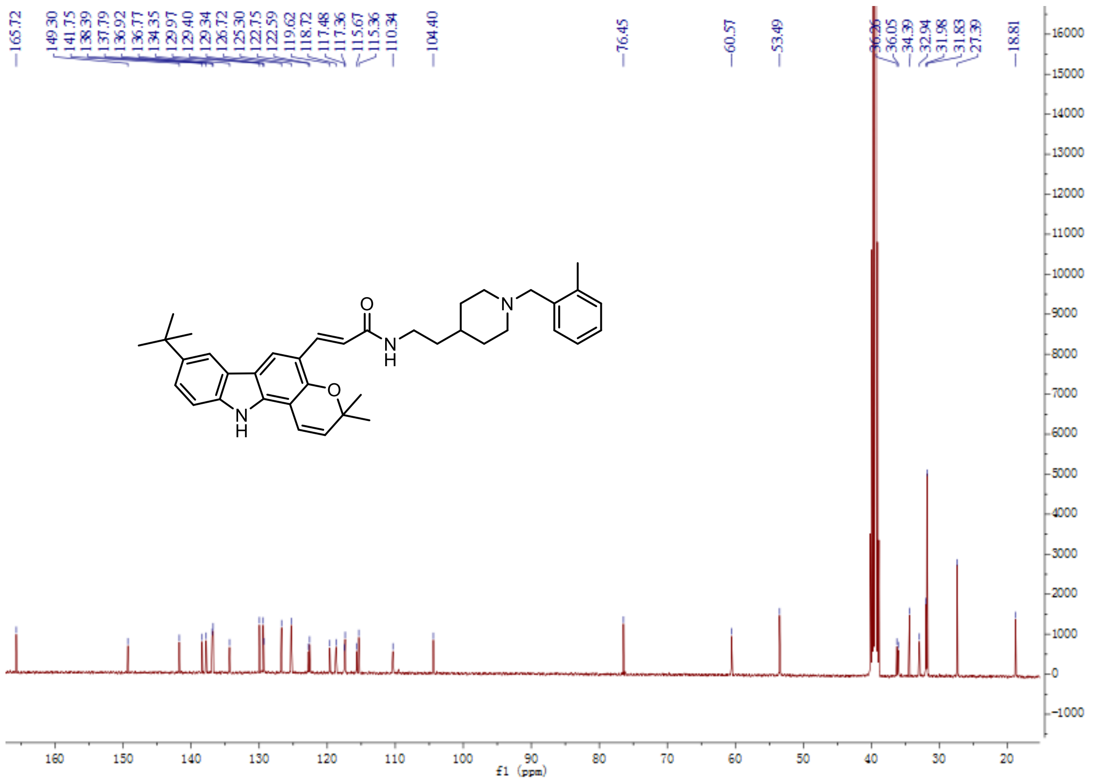

[13] C NMR (DMSO, 400 MHz) Spectrum of Compound **6cc**

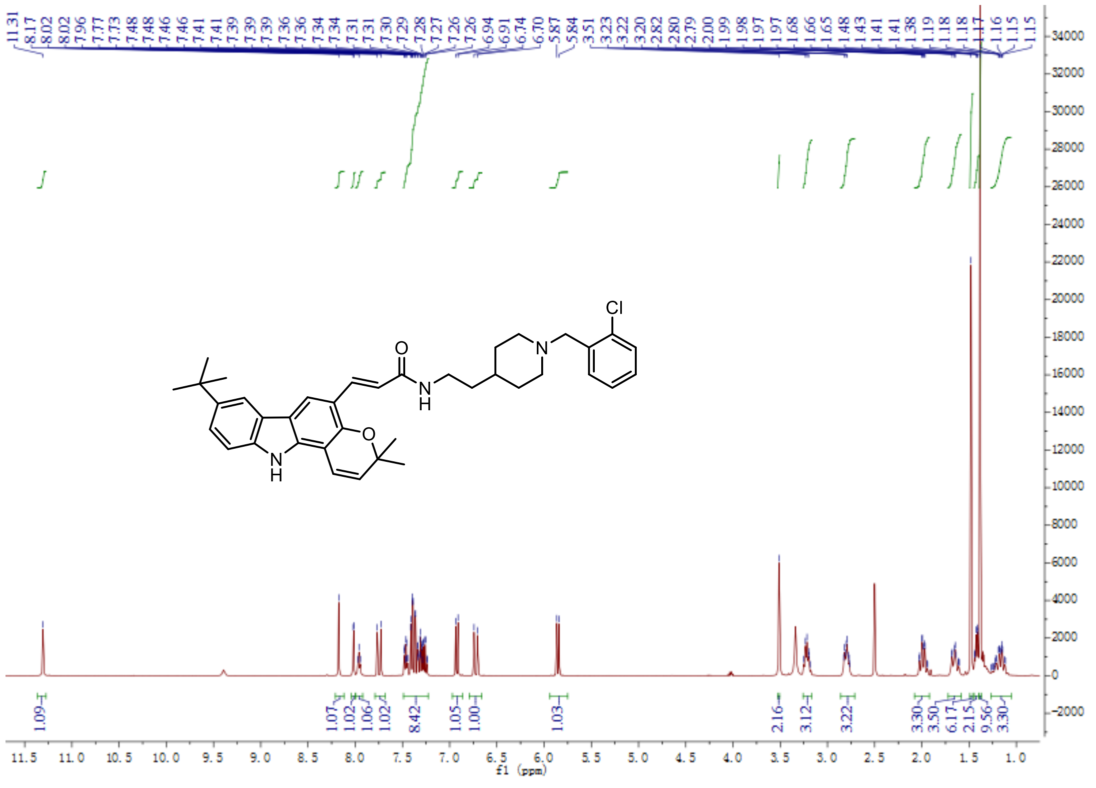

[1] H NMR (DMSO, 400 MHz) Spectrum of Compound **6cd**

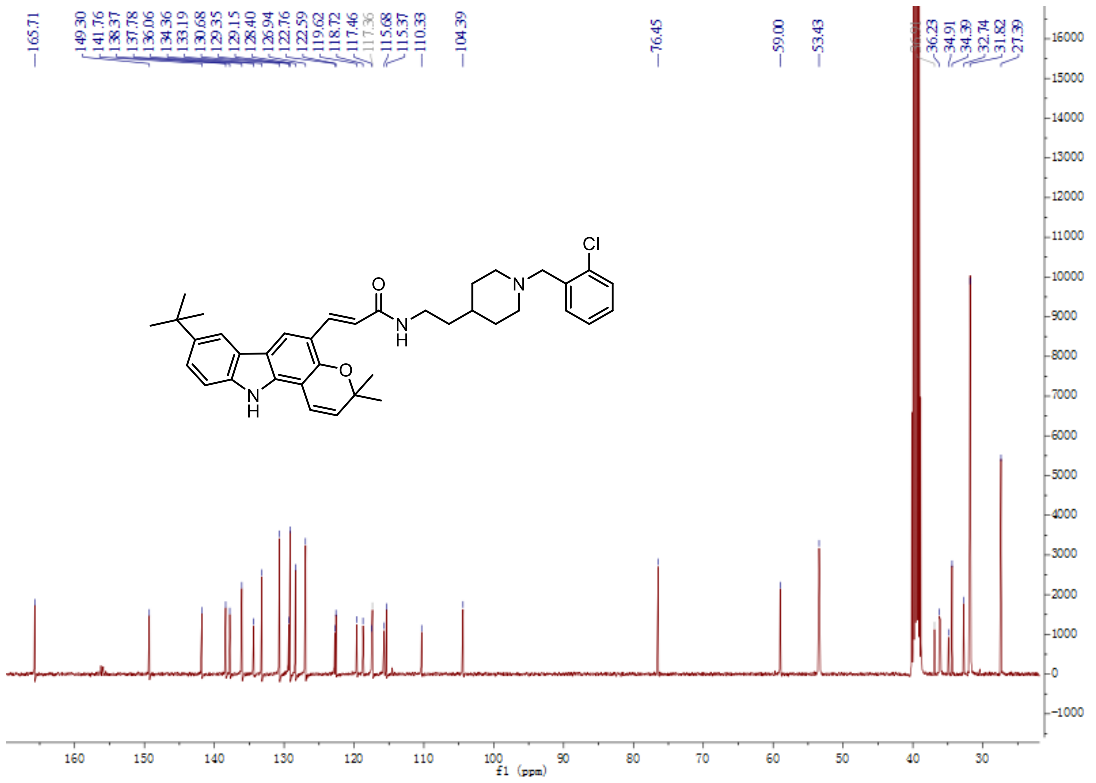

^{13}C NMR (DMSO, 400 MHz) Spectrum of Compound **6cd**

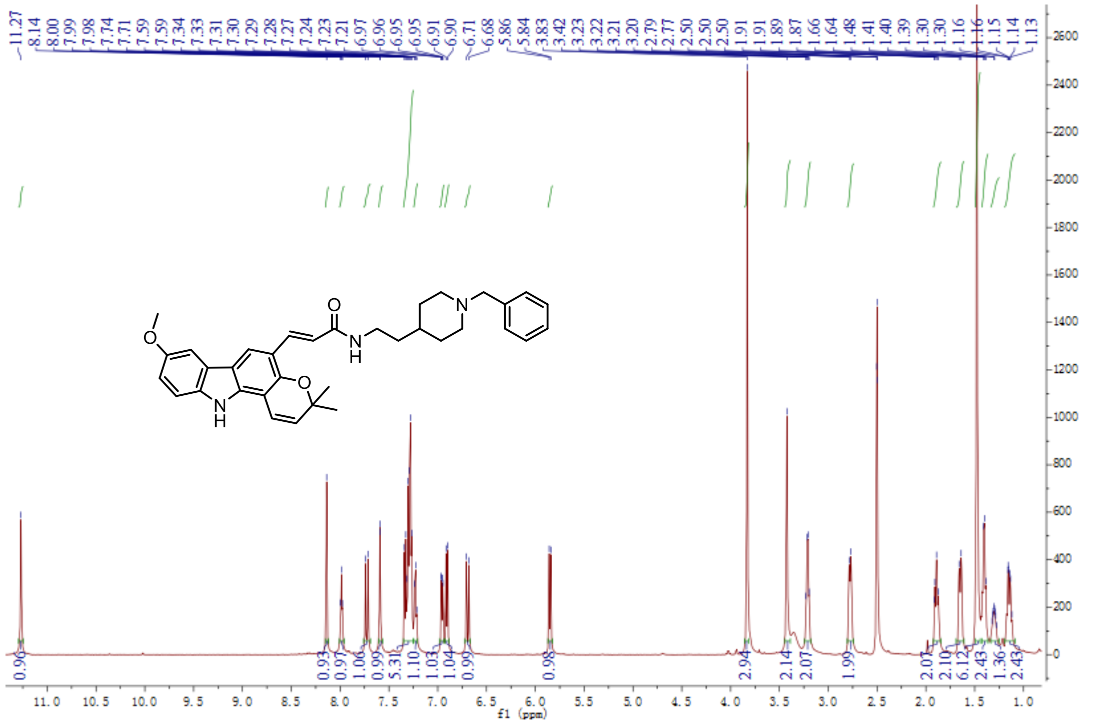

^{1}H NMR (DMSO, 600 MHz) Spectrum of Compound **6ce**

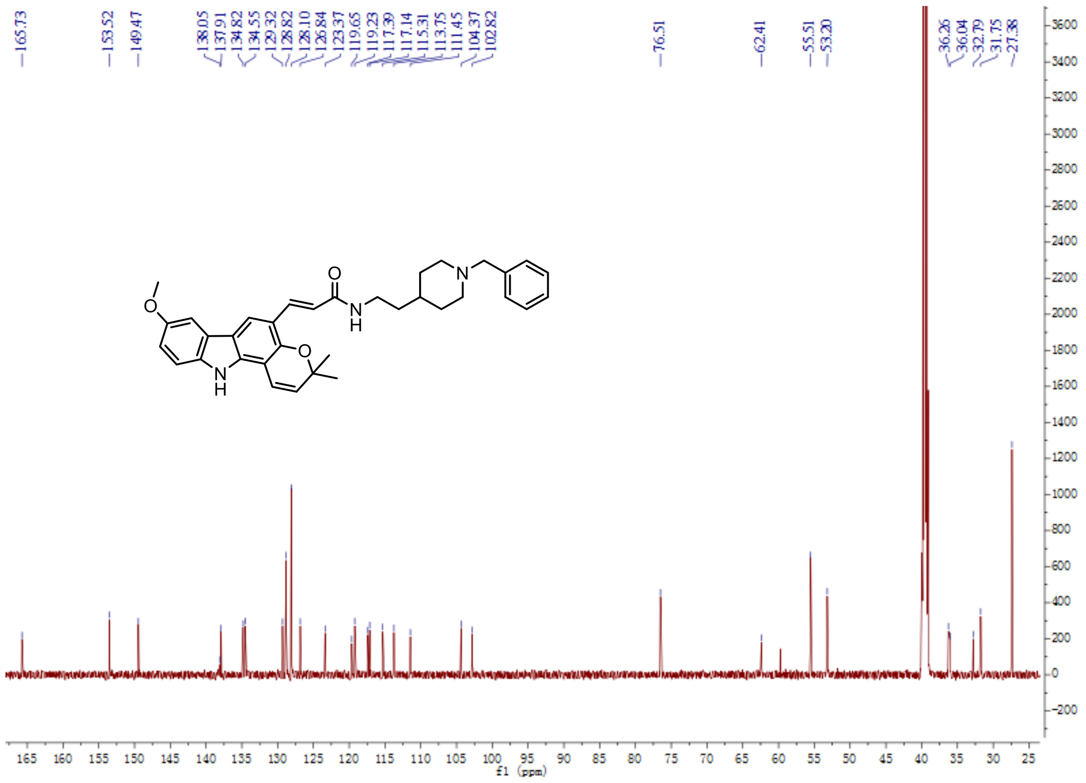

^{13}C NMR (DMSO, 600 MHz) Spectrum of Compound **6ce**

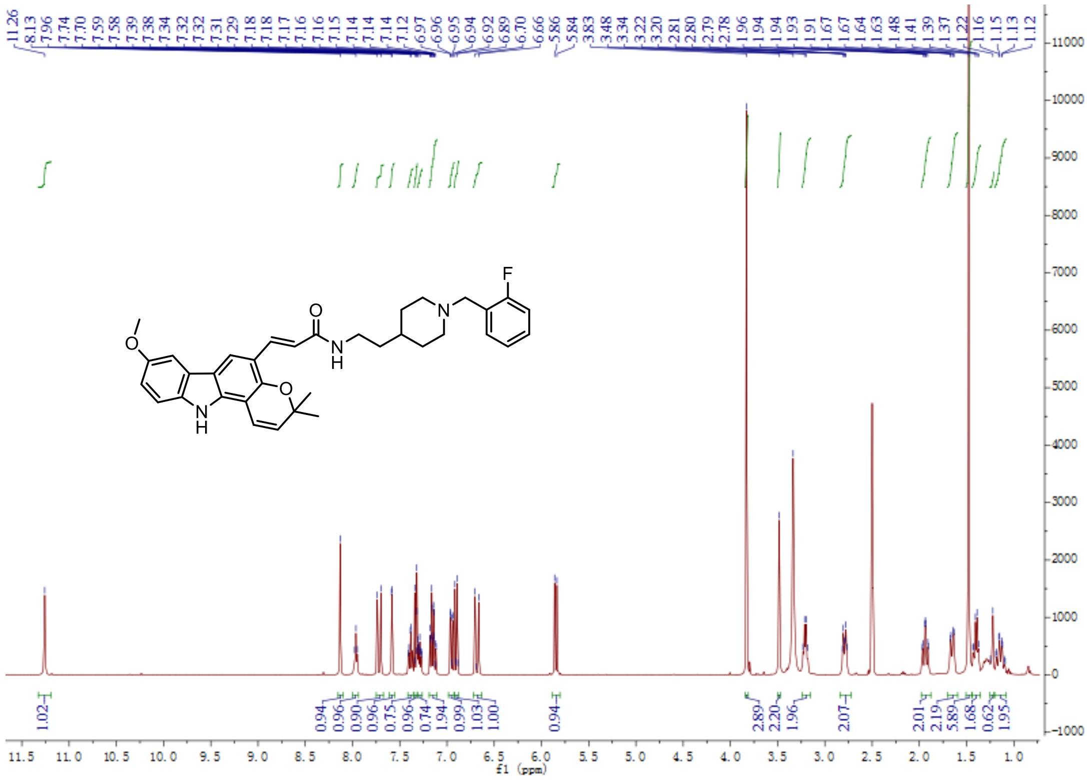

^{1}H NMR (DMSO, 600 MHz) Spectrum of Compound **6cf**

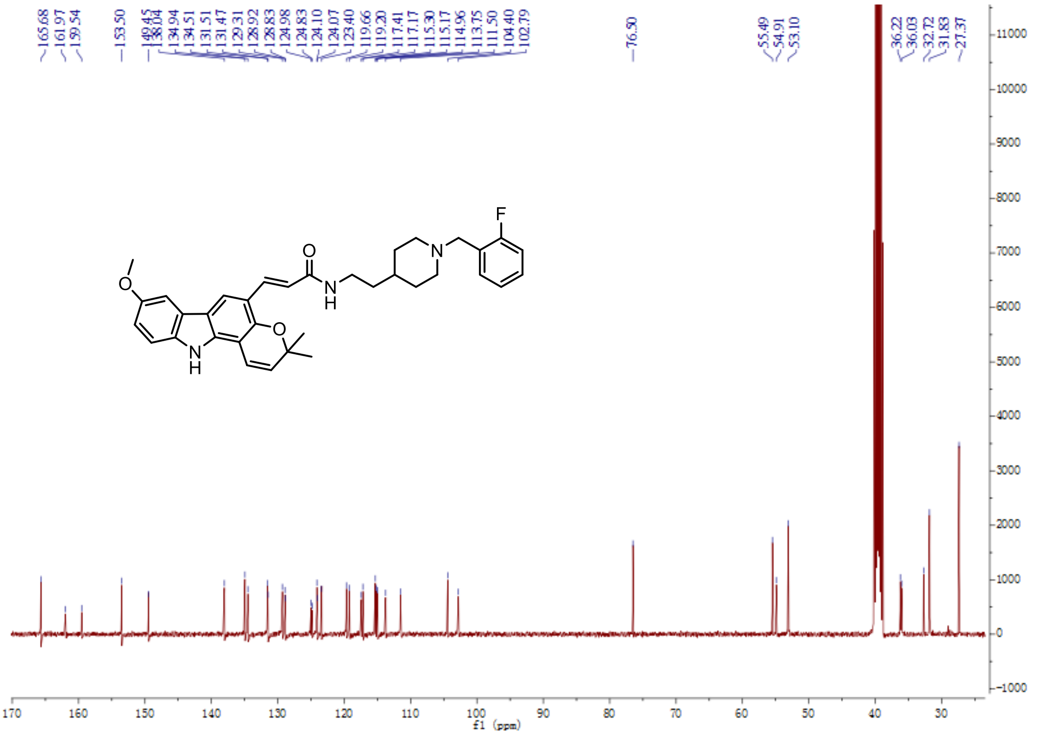

^{13}C NMR (DMSO, 600 MHz) Spectrum of Compound **6cf**

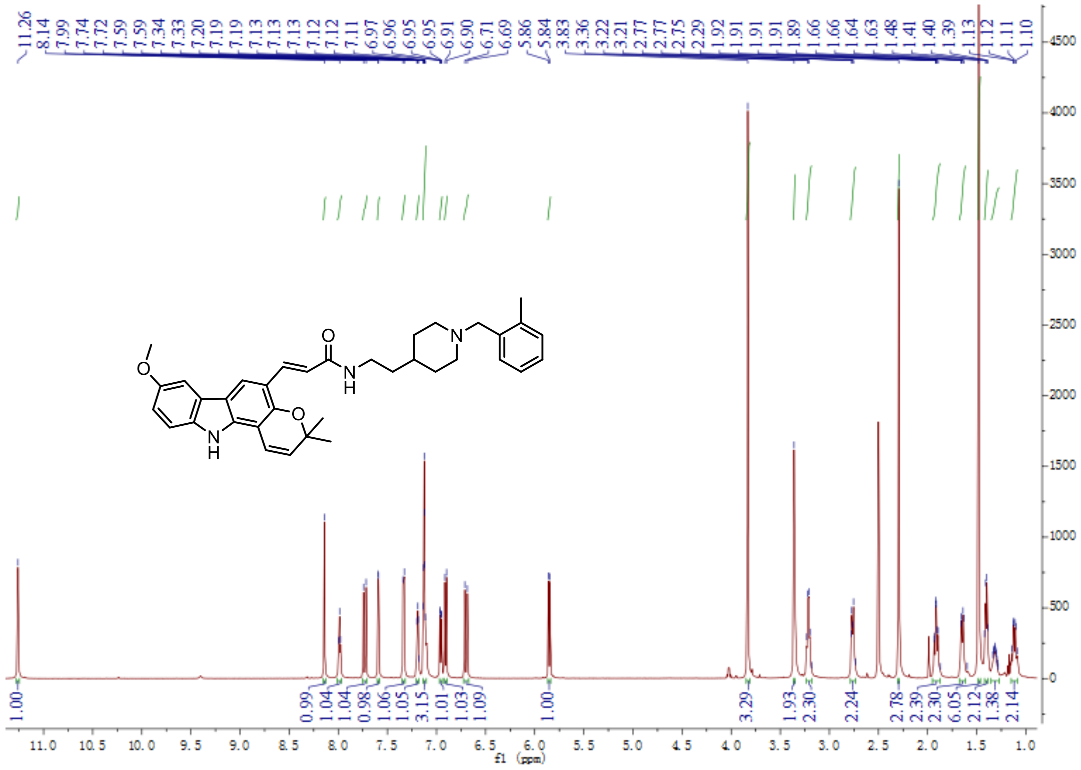

^{1}H NMR (DMSO, 600 MHz) Spectrum of Compound **6cg**

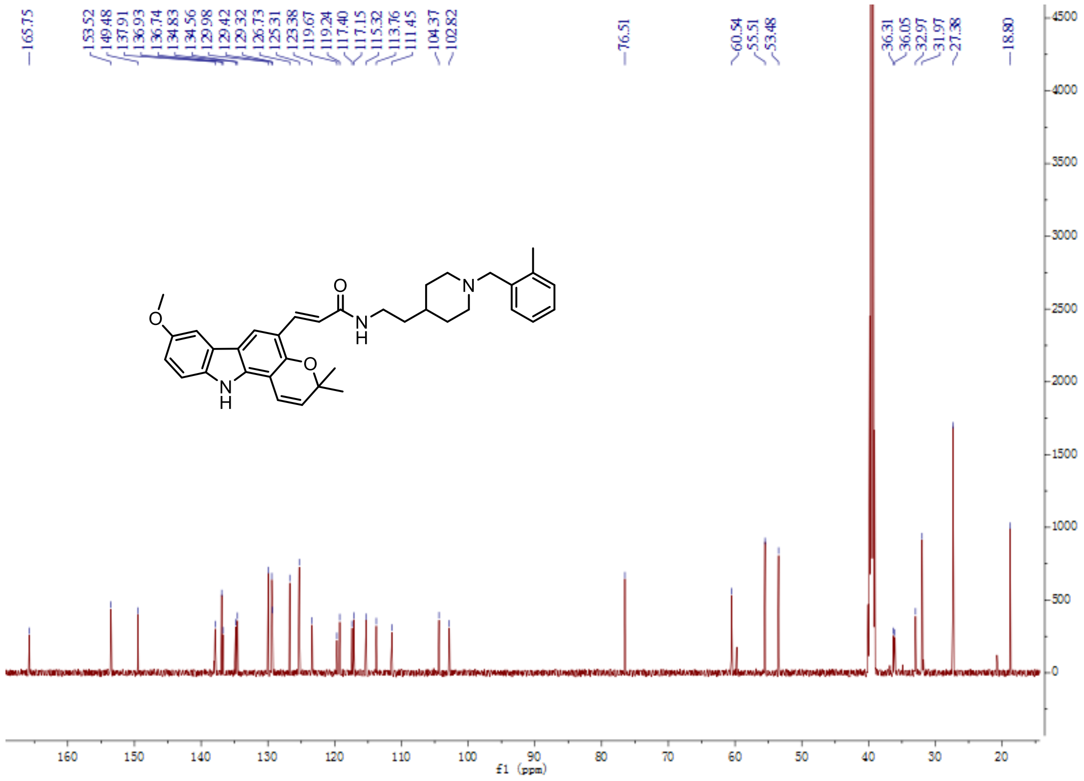

[13] C NMR (DMSO, 600 MHz) Spectrum of Compound **6cg**

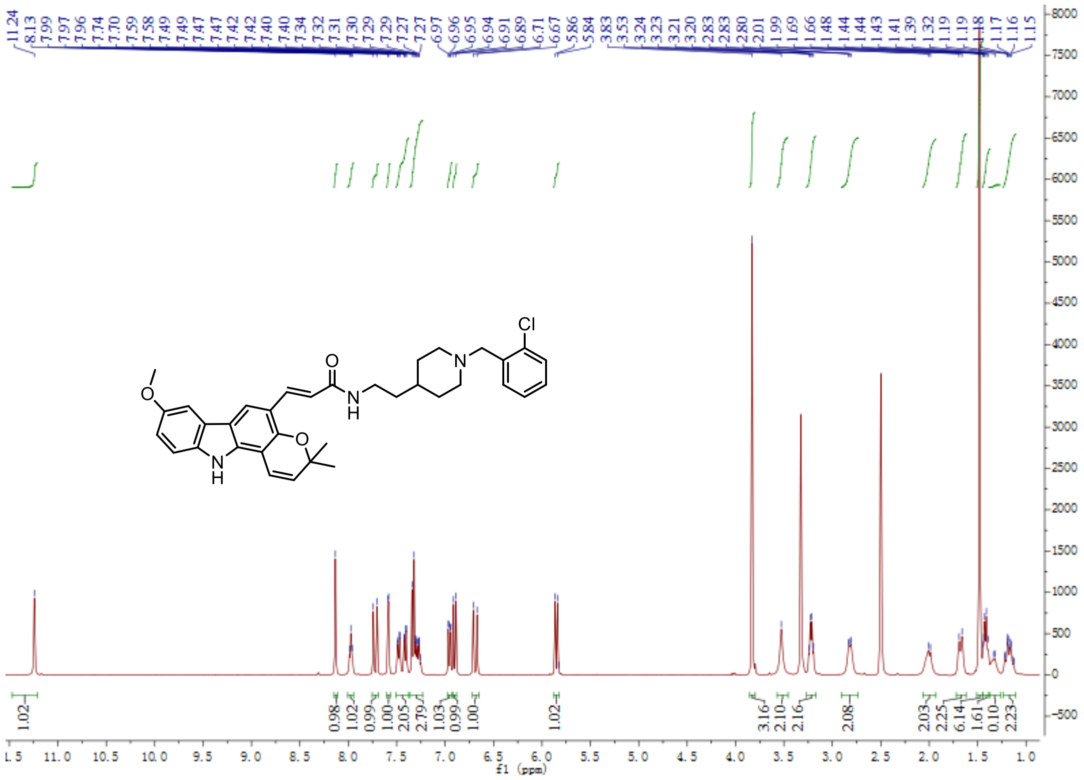

[1] H NMR (DMSO, 600 MHz) Spectrum of Compound **6ch**

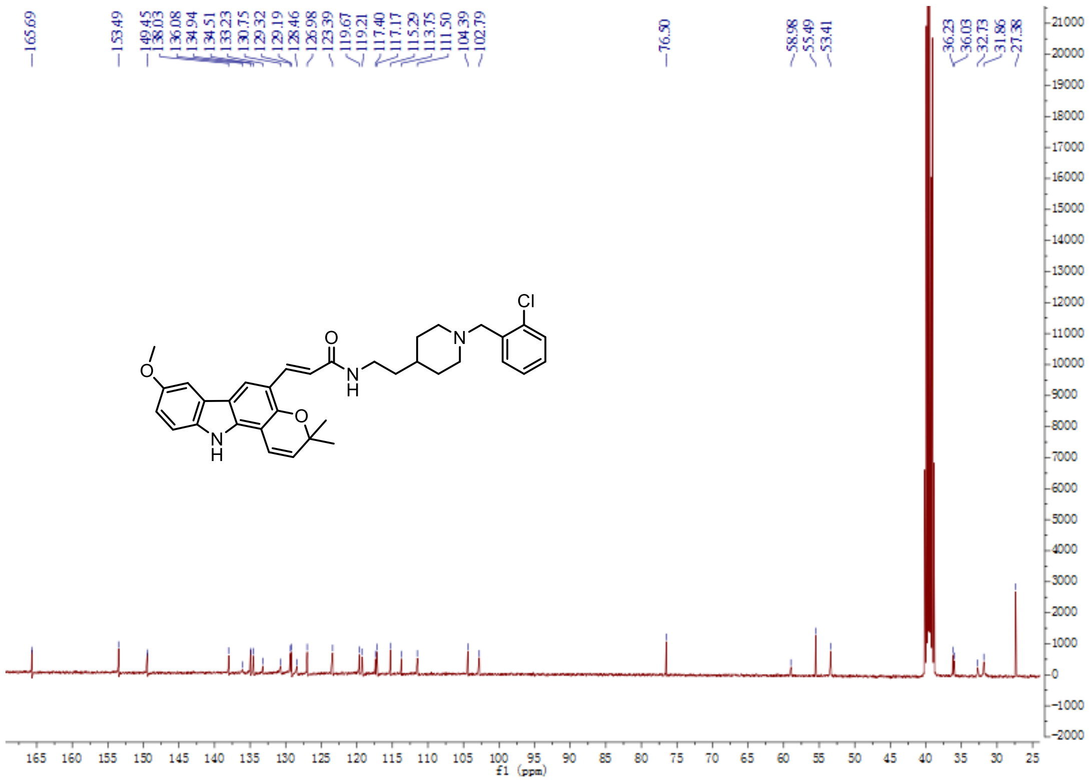

[13] C NMR (DMSO, 600 MHz) Spectrum of Compound **6ch**

2. HPLC analysis of all final compounds

System: Agilent 1100 Series

Mobile phase: methanol / water

Detector: DAD at 254 nm

Column: YMC C18 column (150 mm×4.6 mm, 5μm)

Flow rate: 1 mL/min

Compound	Methanol %	Purity	Retention time (min)
**6aa**	80%	99.11%	6.724
**6ab**	80%	99.68%	15.023
**6ac**	90%	95.89%	6.479

**6ad**	80%	98.56%	17.62
**6ae**	85%	95.28%	4.726
**6af**	85%	95.90%	5.379
**6ag**	85%	98.87%	5.417
**6ah**	85%	97.53%	10.823
**6ba**	85%	99.70%	12.342
**6bb**	80%	98.66%	4.628
**6bc**	80%	99.59%	6.887
**6bd**	80%	98.94%	8.25
**6be**	85%	99.21%	11.41
**6bf**	90%	98.72%	12.924
**6bg**	90%	99.64%	14.598
**6bh**	85%	95.41%	6.744
**6bi**	85%	97.25%	10.984
**6bj**	85%	98.04%	13.025
**6ca**	85%	97.05%	8.074
**6cb**	85%	98.62%	13.255
**6cc**	85%	97.45%	15.597
**6cd**	85%	96.27%	4.759
**6ce**	85%	95.98%	6.179
**6cf**	85%	95.10%	6.68
**6cg**	85%	96.23%	8.576
**6ch**	85%	98.67%	14.652
